# Interaction of genetic variation at *ADH1B* and *MLXIPL* with alcohol consumption for elevated serum urate level and gout among people of European ethnicity

**DOI:** 10.1186/s13075-024-03279-9

**Published:** 2024-02-08

**Authors:** Min H Chuah, Megan P Leask, Ruth K Topless, Gregory D Gamble, Nicholas A Sumpter, Lisa K Stamp, Tony R Merriman, Nicola Dalbeth

**Affiliations:** 1 Department of Medicine, Faculty of Medical and Health Sciences, University of Auckland, 85 Park Rd, Grafton, Auckland, New Zealand; 2 Department of Medicine, University of Alabama at Birmingham, Birmingham, AL USA; 3 Department of Biochemistry, University of Otago, Dunedin, New Zealand; 4 Department of Medicine, University of Otago Christchurch, Christchurch, New Zealand

**Keywords:** Urate, Hyperuricaemia, Gout, Alcohol, Single-nucleotide polymorphism, *ADH1B*

## Abstract

**Background:**

Alcohol consumption is a risk factor for hyperuricaemia and gout. Multiple single-nucleotide polymorphisms (SNPs) have been identified as associated with both alcohol consumption and serum urate or gout in separate genome-wide association studies (GWAS). This study aimed to identify and characterise interactions between these shared signals of genetic association and alcohol consumption for serum urate level, hyperuricaemia, and gout.

**Methods:**

This research was conducted using the UK Biobank resource. The association of alcohol consumption with serum urate and gout was tested among 458,405 European participants. Candidate SNPs were identified by comparing serum urate, gout, and alcohol consumption GWAS for shared signals of association. Multivariable-adjusted linear and logistic regression analyses were conducted with the inclusion of interaction terms to identify SNP-alcohol consumption interactions for association with serum urate level, hyperuricaemia, and gout. The nature of these interactions was characterised using genotype-stratified association analyses.

**Results:**

Alcohol consumption was associated with elevated serum urate and gout. For serum urate level, non-additive interactions were identified between alcohol consumption and rs1229984 at the *ADH1B* locus (*P* = 3.0 × 10^−44^) and rs6460047 at the *MLXIPL* locus (*P* = 1.4 × 10^−4^). *ADH1B* also demonstrated interaction with alcohol consumption for hyperuricaemia (*P* = 7.9 × 10^−13^) and gout (*P* = 8.2 × 10^−9^). Beer intake had the most significant interaction with *ADH1B* for association with serum urate and gout among men, while wine intake had the most significant interaction among women. In the genotype-stratified association analyses, *ADH1B* and *MLXIPL* were associated with serum urate level and *ADH1B* was associated with hyperuricaemia and gout among consumers of alcohol but not non-consumers.

**Conclusions:**

In this large study of European participants, novel interactions with alcohol consumption were identified at *ADH1B* and *MLXIPL* for association with serum urate level and at *ADH1B* for association with hyperuricaemia and gout. The association of *ADH1B* with serum urate and gout may occur through the modulation of alcohol metabolism rate among consumers of alcohol.

**Supplementary Information:**

The online version contains supplementary material available at 10.1186/s13075-024-03279-9.

## Background

Gout is a common form of inflammatory arthritis resulting from the deposition of monosodium urate crystals in and around joints [1]. Hyperuricaemia is a key step in the pathogenesis of gout. Alcohol consumption is strongly associated with elevated serum urate level [2] and incident gout [3–5]. Genome-wide association studies (GWAS) have identified multiple single-nucleotide polymorphisms (SNPs) associated with serum urate or gout [6] and habitual alcohol consumption [7].

Previous studies have reported the contribution of interactions between genetic factors and alcohol consumption to increased risk of hyperuricaemia and gout [8–10]. Several analyses of SNPs at specific candidate genes have identified SNP-alcohol interactions for association with serum urate and gout. In a Taiwanese population, the risk of hyperuricaemia was significantly increased by interaction with alcohol consumption among participants with the urate-raising allele of *ABCG2* rs2231142 in both men and women [11]. Among Japanese males with alcohol dependence, the fast-metabolising variant of *ADH1B* and the inactive variant of *ALDH2* were associated with elevated serum urate level [12]. These variants were also associated with increased risk of gout among Japanese male consumers of alcohol but not non-consumers [13]. Alcohol exposure was found to reduce the genetic risk of gout associated with SNPs at *GCKR* and *A1CF* in New Zealand European individuals [14]. A SNP -alcohol interaction for gout was also observed at *LRP2* among New Zealand Polynesian (Māori or Pacific) people [15]. However, there has not been a systematic analysis of SNPs at all loci identified to be associated with both alcohol consumption and serum urate or gout.

This study aimed to identify and evaluate the nature of interactions between these shared genetic signals of association and alcohol consumption in influencing serum urate level, hyperuricaemia, and gout.

## Methods

### Participants

This research was conducted using the UK Biobank resource (approval number 12611). The UK Biobank is a major biomedical database that recruited more than 500,000 individuals aged 40–69 years from throughout the UK between 2006 and 2010. Upon recruitment, each participant completed a comprehensive baseline demographic, lifestyle, and health questionnaire. Baseline anthropometric and biochemical measurements were also collected. The North West Multi-Centre Research Ethics Committee granted ethical approval for the UK Biobank (REC reference: 16/NW/0274). All participants provided written informed consent for the use of their anonymised data in any health-related research.

Inclusion criteria for this study consisted of self-reported European ethnicity (white, British, Irish, or any other white background) and available genotyping data for the genetic analyses. Participants who had withdrawn from the UK Biobank or with genotyping quality control failure were excluded.

### Serum urate level and gout status

Baseline serum urate level in the UK Biobank was measured by uricase-PAP (peroxidase/aminophenazone/phenol) analysis on a Beckman Coulter AU5800. In the present study, hyperuricaemia was defined as a serum urate level of ≥410 μmol/L (0.41 mmol/L; 7.38 mg/dL) for both men and women [14]. Participants taking urate-lowering therapy were excluded from the urate analyses. Gout cases were identified using a validated case definition of self-reported gout or urate-lowering therapy use (allopurinol or sulphinpyrazone) with no previous hospital diagnosis of leukaemia or lymphoma based on the International Classification of Diseases, 10^th^ Revision, code C81-C96 [16]. Although febuxostat was included in the original definition for urate-lowering therapy use [16], this was excluded from the present study as it was not used by any participants in the UK Biobank.

### Alcohol consumption and other dietary intake

Self-reported alcohol consumption data were obtained through the UK Biobank baseline Touchscreen Questionnaire. All participants who indicated that they consume alcohol were asked to quantify their weekly or monthly intake of beer/cider (pints), spirits/liqueur (measures), red wine (glasses), white wine/champagne (glasses), fortified wine (glasses), and other alcoholic beverages (glasses). A standardised number of UK alcohol units was applied to each alcoholic drink to calculate the weekly units of alcohol consumed. Values greater than zero were classified as any alcohol intake. Further details for the calculation and standardisation of weekly alcohol intake data are available in Additional file 1: Supplementary Methods.

Other dietary intake values were determined from the responses provided for daily or weekly food intake frequency in the baseline Touchscreen Questionnaire (Additional file 1: Supplementary Methods).

### Estimated glomerular filtration rate

Estimated glomerular filtration rate (eGFR) was used as the marker for kidney function in the present study. eGFR was calculated according to the 2009 Chronic Kidney Disease Epidemiology Collaboration (CKD-EPI) equation based on serum creatinine [17].

### Genotyping and selection of SNPs for analysis

UK Biobank genotyping was conducted using the Affymetrix UK Biobank Axiom array (825,927 markers) or Affymetrix UK BiLEVE Axiom array (807,411 markers) [18]. Genetic data were phased using SHAPEIT3 and imputed to ~96 million SNPs using IMPUTE4, with a combined reference panel from the Haplotype Reference Consortium, UK10K, and 1000 Genomes (phase 3) [18].

GWAS of habitual alcohol consumption published within 5 years before the commencement of this study (Additional file 1: Table S1) were compared with the Tin et al. [6] serum urate and gout GWAS to identify shared (co-localised) signals of association at genome-wide significance. For each overlapping signal, a LocusZoom plot [19] was created within the UK Biobank for the shared SNP that was most strongly associated with serum urate or gout in the Tin et al. [6] GWAS. Only the lead shared SNP at each locus was selected for interaction analysis.

### Statistical analysis

All statistical analyses were conducted using the IBM SPSS Statistics (network) software, version 27.0.0.0 (IBM, New York, USA). Both combined gender (self-reported) and gender-stratified analyses were performed. Bonferroni correction for multiple testing was applied to determine experiment-wide significance in the fully adjusted models.

Multivariable linear and logistic regression analyses were used to assess the association of alcohol consumption with serum urate level, hyperuricaemia, and gout. Each analysis was adjusted for demographic features (age, gender (all participants only), Townsend deprivation index), comorbidities (body mass index (BMI), diuretics use, estimated glomerular filtration rate (eGFR), high cholesterol, hypertension, cardiac problems, peripheral vascular disease, stroke, diabetes mellitus, smoking status), and weekly dietary intake (other alcoholic beverage types (beer/spirits/wine/other) if applicable, meat, fish, coffee, tea, fruit, vegetables, bread, cereal, cheese). Only the analyses of beer, spirits, or wine intake included adjustment for the other alcoholic beverage types.

For the interaction analysis, multivariable linear and logistic regression were used to calculate interaction terms between each SNP (as a binarized dominant model of presence of urate-increasing or gout risk allele vs. absence of allele) and alcohol consumption (as a continuous variable of units per week) for association with serum urate level, hyperuricaemia, and gout. Any experiment-wide significant SNP-alcohol consumption interactions were also tested for the other phenotypes and further stratified by alcoholic beverage type (beer, spirits, and wine intake). All analyses were adjusted as described previously.

Genotype-stratified association analysis was used to characterise the nature of significant genetic interactions between alcohol consumption (all alcohol, beer, spirits, and wine intake) and serum urate level, hyperuricaemia, and gout. Participants were stratified by binarized genotype (presence of urate-increasing or gout risk allele vs. absence of allele) and binarized alcohol consumption (any alcohol vs. no alcohol) into four groups. Using non-consumers of alcohol without the urate-increasing or gout risk allele as the reference group, beta values for serum urate level and odds ratios for hyperuricaemia and gout were calculated in each group using multivariable linear and logistic regression. *P*-values for the serum urate level difference (beta value) and odds ratio of hyperuricaemia or gout between genotype groups in only the alcohol-exposed subset of participants (*P*_difference_) were also calculated. All analyses were adjusted as described previously.

## Results

### Participant characteristics

Data for 458,405 eligible participants were extracted from the UK Biobank. 431,555 participants were eligible for the serum urate analyses and 458,367 participants for the gout analyses (451,144 controls; 7,223 gout cases). The baseline demographic and clinical characteristics of the study participants are presented in Table 1. Gender-stratified baseline characteristics for all eligible participants are shown separately in Additional file 1: Table S2.

**Table 1:** Baseline demographic and clinical characteristics of study population. Data shown as mean (standard deviation) unless indicated otherwise

	**All eligible participants**	**Participants in urate analysis**	**Participants in gout analysis**	
			**Controls**	**Gout cases**
*N (%)*	458,405	431,555 (94.1%)	451,144 (98.4%)	7223 (1.6%)
Men, *N* (%)	209,524 (45.7%)	195,068 (45.2%)	202,829 (45.0%)	6664 (92.3%)
Age (years)	56.77 (8.03)	56.71 (8.03)	56.71 (8.03)	60.00 (6.87)
Townsend deprivation index	−1.46 (2.99)	−1.46 (2.99)	−1.46 (2.99)	−1.17 (3.11)
Gout, *N* (%)	7223 (1.6%)	1924 (0.4%)	-	-
Urate-lowering therapy use, *N* (%)	5244 (1.1%)	-	-	5206 (72.1%)
Serum urate level (μmol/L)	309.08 (80.41)	308.76 (80.30)	308.06 (79.60)	372.61 (102.32)
Hyperuricaemia (≥410 μmol/L), *N* (%)	48,563 (11.1%)	47,641 (11.0%)	46,099 (10.7%)	2455 (35.7%)
Serum creatinine level (μmol/L)	72.24 (17.89)	72.03 (17.11)	71.97 (17.04)	88.79 (43.25)
eGFR (mL/min/1.73m^2^)	90.52 (13.26)	90.62 (13.16)	90.64 (13.15)	83.37 (17.51)
Diuretics, *N* (%)	38,129 (8.3%)	35,379 (8.2%)	36,928 (8.2%)	1186 (16.4%)
Body mass index (kg/m^2^)	27.39 (4.77)	27.35 (4.74)	27.34 (4.75)	30.72 (4.96)
High cholesterol,* N* (%)	56,201 (12.3%)	51,918 (12.0%)	54,166 (12.0%)	2030 (28.1%)
Hypertension, *N* (%)	118,698 (25.9%)	110,047 (25.5%)	114,565 (25.4%)	4110 (56.9%)
Cardiac problem, *N* (%)	1466 (0.3%)	1364 (0.3%)	1411 (0.3%)	55 (0.8%)
Peripheral vascular disease, *N* (%)	865 (0.2%)	822 (0.2%)	855 (0.2%)	10 (0.1%)
Stroke, *N* (%)	6062 (1.3%)	5564 (1.3%)	5804 (1.3%)	252 (3.5%)
Diabetes, *N* (%)	17,995 (3.9%)	16,434 (3.8%)	17,139 (3.8%)	853 (11.8%)
Current smoker, *N* (%)	47,745 (10.5%)	45,030 (10.5%)	47,086 (10.5%)	654 (9.1%)
Previous smoker, *N* (%)	162,593 (35.6%)	152,406 (35.4%)	159,004 (35.4%)	3569 (49.6%)
Never smoked, *N* (%)	246,433 (54.0%)	232,590 (54.1%)	243,446 (54.2%)	2974 (41.3%)
Current alcohol drinker, *N* (%)	427,252 (93.3%)	402,182 (93.3%)	420,330 (93.3%)	6890 (95.5%)
Previous alcohol drinker, *N* (%)	15,944 (3.5%)	15,001 (3.5%)	15,712 (3.5%)	227 (3.1%)
Never alcohol drinker, *N* (%)	14,797 (3.2%)	13,981 (3.2%)	14,698 (3.3%)	98 (1.4%)
All alcohol intake (units per week)	19.44 (19.85)	19.28 (19.64)	19.18 (19.56)	34.54 (29.00)
Beer intake (units per week)	6.37 (13.11)	6.21 (12.82)	6.13 (12.70)	19.94 (24.24)
Spirits intake (units per week)	1.54 (4.87)	1.53 (4.80)	1.52 (4.79)	2.69 (8.01)
All wine intake (units per week)	11.53 (14.35)	11.54 (14.33)	11.52 (14.29)	11.96 (17.56)
Meat intake (servings per week)	5.53 (2.71)	5.52 (2.71)	5.52 (2.70)	6.54 (2.73)
Fish intake (servings per week)	2.23 (1.57)	2.27 (1.57)	2.27 (1.57)	2.35 (1.62)
Coffee intake (cups per week)	14.46 (14.72)	14.49 (14.72)	14.50 (14.74)	12.25 (13.02)
Tea intake (cups per week)	24.24 (20.28)	24.26 (20.28)	24.26 (20.28)	22.68 (20.30)
Fruit intake (pieces per week)	21.30 (17.50)	21.33 (17.54)	21.34 (17.51)	19.09 (16.77)
Vegetable intake (tablespoons per week)	33.96 (22.28)	33.96 (22.18)	33.96 (22.25)	34.03 (24.04)
Bread intake (slices per week)	12.44 (8.62)	12.42 (8.62)	12.41 (8.61)	14.57 (9.11)
Cereal intake (bowls per week)	4.58 (2.78)	4.58 (2.78)	4.59 (2.78)	3.90 (2.81)
Cheese intake (servings per week)	2.42 (1.76)	2.43 (1.77)	2.43 (1.77)	2.28 (1.69)

In the serum urate analysis cohort, 11.0% of participants had hyperuricaemia. Men had a higher mean serum urate level and prevalence of hyperuricaemia (354.7 µmol/L and 20.6%, respectively) than women (270.7 µmol/L and 3.1%, respectively). In the gout analysis cohort, participants with gout were on average 3.3 years older, predominantly men (92.3% of cases), had a higher prevalence of nearly all included comorbidities, and were less likely to be current smokers (9.1% of cases).

The data for all alcohol consumption among all participants were normally distributed (Additional file 1: Figure S1). Men were more likely to be current alcohol consumers than women (94.9% of men compared to 91.9% of women) and consumed larger amounts of alcohol (23.2 units/week compared to 13.8 units/week of all alcohol), particularly beer (16.8 units/week compared to 4.1 units/week of beer).

### Association of alcohol consumption with serum urate level and hyperuricaemia

All alcohol, beer, spirits, and wine intake were associated with increased serum urate level and hyperuricaemia in all groups (Table 2 and Additional file 1: Table S3). Among all participants, beer intake was associated with the largest μmol/L increase in serum urate level for each unit consumed per week (*β* 0.75, SE 0.01, *P* < 1.0 × 10^−300^), followed by spirits (β 0.45, SE 0.02, *P* < 1.0 × 10^−300^), then wine (*β* 0.33, SE 0.01, *P* = 2.8 × 10^−199^). This pattern was also demonstrated among men and women separately. The same trends were observed among all participants, men, and women for the association of alcohol consumption with hyperuricaemia.

**Table 2:** Association of alcohol intake phenotypes with serum urate level, hyperuricaemia, and gout

**Group**	**Alcohol intake (units/week)**	**Association with serum urate (μmol/L)**				**Association with hyperuricaemia**			**Association with gout**		
		***β***	**SE**	***Β***	***P*-value**	**OR**	**95% CI**	***P*-value**	**OR**	**95% CI**	***P*-value**
All participants		*N* = 345,339				*N* = 345,339			*N* = 350,057		
	All alcohol	0.49	0.01	0.12	< 1.00 × 10^−300^	1.015	1.014, 1.015	< 1.00 × 10^−300^	1.013	1.013, 1.014	1.16 × 10^−173^
	Beer	0.75	0.01	0.12	< 1.00 × 10^−300^	1.022	1.021, 1.023	< 1.00 × 10^−300^	1.023	1.021, 1.024	3.86 × 10^−285^
	Spirits	0.45	0.02	0.03	1.41 × 10^−104^	1.013	1.011, 1.016	9.14 × 10^−39^	1.006	1.002, 1.010	0.001
	Wine	0.33	0.01	0.06	< 1.00 × 10^−300^	1.009	1.008, 1.010	6.29 × 10^−22^	1.004	1.002, 1.006	3.62 × 10^−7^
Men		*N* = 163,469				*N* = 163,469			*N* = 167,653		
	All alcohol	0.51	0.01	0.16	< 1.00 × 10^−300^	1.015	1.014, 1.015	< 1.00 × 10^−300^	1.014	1.013, 1.015	7.37 × 10^−172^
	Beer	0.74	0.01	0.17	< 1.00 × 10^−300^	1.021	1.021, 1.022	< 1.00 × 10^−300^	1.023	1.021, 1.024	2.04 × 10^−281^
	Spirits	0.40	0.03	0.03	1.74 × 10^−51^	1.011	1.009, 1.014	1.99 × 10^−27^	1.006	1.002, 1.009	0.002
	Wine	0.31	0.01	0.07	8.33 × 10^−217^	1.009	1.008, 1.009	1.23 × 10^−103^	1.004	1.002, 1.006	6.79 × 10^−7^
Women		*N* = 181,870				*N* = 181,870			*N* = 182,404		
	All alcohol	0.47	0.01	0.10	< 1.00 × 10^−300^	1.018	1.016, 1.020	3.71 × 10^−76^	1.010	1.004, 1.017	0.002
	Beer	0.95	0.03	0.06	1.26 × 10^−197^	1.041	1.036, 1.046	1.55 × 10^−53^	1.029	1.012, 1.045	7.05 × 10^−4^
	Spirits	0.77	0.04	0.04	4.88 × 10^−103^	1.031	1.026, 1.037	5.31 × 10^−30^	1.021	1.006, 1.038	0.01
	Wine	0.40	0.01	0.08	< 1.00 × 10^−300^	1.013	1.011, 1.015	3.53 × 10^−33^	1.006	0.998, 1.014	0.12

*Abbreviations*: *β* unstandardised beta coefficient per unit/week increase in alcohol intake, *SE* Otandard error, *B* standardised beta coefficient per unit/week increase in alcohol intake, *OR* Odds ratio per unit/week increase in alcohol intake, *95% CI *95% confidence interval

Adjusted for age, gender (all participants only), Townsend deprivation index, BMI, diuretics use, eGFR, high cholesterol, hypertension, cardiac problem, peripheral vascular disease, stroke, diabetes mellitus, smoking status, other alcoholic beverage types (beer/spirits/wine/other) intake (excluding all alcohol intake analyses), meat intake, fish intake, coffee intake, tea intake, fruit intake, vegetable intake, bread intake, cereal intake, and cheese intake

Bonferroni-corrected experiment-wide significance for each analysis: *P* < 0.01

### Association of alcohol consumption with gout

All alcohol and beer intake were associated with gout in all groups (Table 2; and Additional file 1: Table S3). However, spirits and wine intake were not significantly associated with gout in women. Among all participants, each unit of beer consumed per week had the largest association with gout (OR 1.023, 95% CI 1.021–1.024, *P* = 3.9 × 10^−285^), followed by spirits (OR 1.006, 95% CI 1.002–1.010, *P* = 0.001) and wine (OR 1.004, 95% CI 1.002–1.006, *P* = 3.6 × 10^−7^). A similar pattern was seen among men and women separately.

### Interaction analysis for serum urate level and hyperuricaemia

Five SNPs were included in the interaction analysis for serum urate level and hyperuricaemia. LocusZoom plots for the five serum urate loci in the UK Biobank are shown in Additional file 1: Figure S2. The serum urate and alcohol consumption GWAS association details for each lead shared SNP are shown in Additional file 1: Table S4. Although not associated with serum urate at genome-wide significance in the Tin et al. [6] GWAS, the *ADH1B* locus was included in the serum urate and hyperuricaemia LocusZoom plots and interaction analysis after significant interactions with alcohol consumption were identified for gout.

Among all participants, significant interactions with alcohol intake as a continuous variable (units per week) were observed for association with serum urate level at rs1229984 in the *ADH1B* locus (*P* = 3.0 × 10^−44^) and rs6460047 in the *MLXIPL* locus (*P* = 1.4 × 10^−4^) (Table 3; and Additional file 1: Table S5), and for association with hyperuricaemia at *ADH1B* rs1229984 only (*P* = 7.9 × 10^−13^) (Table 4; and Additional file 1: Table S6). In the gender-stratified analysis, there was evidence for significant interaction of alcohol intake with *ADH1B* rs1229984 for association with serum urate level and hyperuricaemia among both men (*P* = 1.1 × 10^−17^ and *P* = 4.3 × 10^−9^, respectively) and women (*P* = 1.4 × 10^−26^ and *P* = 1.1 × 10^−6^, respectively). However, the interaction at *MLXIPL* rs6460047 for association with serum urate level was only significant among men (*P* = 0.002).

**Table 3:** Interaction terms between urate-associated SNPs and all alcohol intake (units/week) for serum urate level (μmol/L). Significant interactions highlighted in bold

**Locus**	**SNP**	**Urate-raising allele**	**All participants (*N* = 431,555)**				**Men (*N* = 195,068)**				**Women (*N* = 236,487)**			
			**Obs, %**	**IT (*β*)**	**SE**	***P*-value**	**Obs, %**	**IT (*β*)**	**SE**	***P*-value**	**Obs, %**	**IT (*β*)**	**SE**	***P*-value**
*GCKR*	rs1260326	T	80.0%	0.003	0.010	0.76	83.8%	1.73 × 10^−5^	0.014	0.99	76.9%	−0.035	0.018	0.06
*KLB*	rs11940694	G	77.6%	−0.015	0.014	0.29	81.3%	−0.017	0.019	0.36	74.5%	−0.032	0.026	0.22
***ADH1B***	**rs1229984**	**T**	**80.0%**	**0.408**	**0.029**	**3.02** ×**10**^−**44**^	**83.8%**	**0.336**	**0.039**	**1.05** × **10**^−**17**^	**76.9%**	**0.543**	**0.051**	**1.36** × **10**^−**26**^
***MLXIPL***	**rs6460047**	**T**	**79.6%**	**0.092**	**0.024**	**1.40** × **10**^−**4**^	**83.3%**	**0.098**	**0.032**	**0.002**	76.5%	0.070	0.045	0.12
*NFAT5*	rs113441031	T	79.5%	0.015	0.011	0.15	83.2%	0.004	0.014	0.80	76.4%	0.025	0.019	0.20

*Abbreviations*: *SNP* Single-nucleotide polymorphism, *Obs* Observations (%), *IT* Interaction term for SNP (≥one urate-raising allele vs. none) × all alcohol intake (units/week), *β* unstandardised beta coefficient, *SE* Standard error

Adjusted for age, gender (all participants only), Townsend deprivation index, BMI, diuretics use, eGFR, high cholesterol, hypertension, cardiac problem, peripheral vascular disease, stroke, diabetes mellitus, smoking status, meat intake, fish intake, coffee intake, tea intake, fruit intake, vegetable intake, bread intake, cereal intake, and cheese intake

Although not associated with serum urate levels at genome-wide significance in the Tin et al. [6] GWAS, the *ADH1B* locus was included in the interaction analysis for serum urate after significant interactions with alcohol consumption were identified for gout

Bonferroni-corrected experiment-wide significance for each analysis: *P* < 0.01

**Table 4:** Interaction terms between urate-associated SNPs and all alcohol intake (units/week) for hyperuricaemia. Significant interactions highlighted in bold

**Locus**	**SNP**	**Urate-raising allele**	**All participants (*N* = 431,555)**				**Men (*N* = 195,068)**				**Women (*N* = 236,487)**			
			**Obs, %**	**IT (OR)**	**95% CI**	***P*-value**	**Obs, %**	**IT (OR)**	**95% CI**	***P*-value**	**Obs, %**	**IT (OR)**	**95% CI**	***P*-value**
*GCKR*	rs1260326	T	80.0%	1.000	0.998, 1.001	0.34	83.8%	1.000	0.999, 1.001	0.69	76.9%	0.998	0.995, 1.002	0.28
*KLB*	rs11940694	G	77.6%	1.000	0.998, 1.001	0.76	81.3%	1.000	0.999, 1.002	0.89	74.5%	0.994	0.990, 0.999	0.03
***ADH1B***	**rs1229984**	**T**	**80.0%**	**1.010**	**1.008, 1.013**	**7.88** × **10**^−**13**^	**83.8%**	**1.009**	**1.006, 1.012**	**4.29** × **10**^−**9**^	**76.9%**	**1.021**	**1.013, 1.030**	**1.06** × **10**^−**6**^
*MLXIPL*	rs6460047	T	79.6%	1.003	1.001, 1.006	0.01	83.3%	1.003	1.000, 1.006	0.03	76.5%	1.012	1.001, 1.022	0.03
*NFAT5*	rs113441031	T	79.5%	1.000	0.999, 1.001	0.94	83.2%	1.000	0.999, 1.001	0.56	76.4%	1.003	0.999, 1.006	0.15

*Abbreviations*: *SNP* Single-nucleotide polymorphism, *Obs* Observations (%), *IT* Interaction term for SNP (≥one urate-raising allele vs. none) × all alcohol intake (units/week), *OR* Odds ratio, *95% CI* 95% confidence interval

Adjusted for age, gender (all participants only), Townsend deprivation index, BMI, diuretics use, eGFR, high cholesterol, hypertension, cardiac problem, peripheral vascular disease, stroke, diabetes mellitus, smoking status, meat intake, fish intake, coffee intake, tea intake, fruit intake, vegetable intake, bread intake, cereal intake, and cheese intake

Although not associated with serum urate levels at genome-wide significance in the Tin et al. [6] GWAS, the *ADH1B* locus was included in the interaction analysis for hyperuricaemia after significant interactions with alcohol consumption were identified for gout

Bonferroni-corrected experiment-wide significance for each analysis: *P* < 0.01

After stratifying by alcoholic beverage type, beer intake demonstrated the most significant interactions with *ADH1B* rs1229984 among men for association with serum urate level (Additional file 1: Table S7) and hyperuricaemia (Additional file 1: Table S8), while wine intake demonstrated the most significant interactions among women. For association with serum urate level, beer intake also showed the most significant interaction with *MLXIPL* rs6460047 among men (Additional file 1: Table S7).

### Interaction analysis for gout

Four SNPs were included in the interaction analysis for gout. LocusZoom plots for the four gout loci in the UK Biobank are shown in Additional file 1: Figure S3. The association details from the gout and alcohol consumption GWAS for each lead shared SNP are shown in Additional file 1: Table S4.

Only *ADH1B* rs1229984 demonstrated significant interaction with continuous alcohol intake for association with gout among all participants (*P* = 8.2 × 10^−9^) (Table 5; and Additional file 1: Table S9). This interaction remained significant among men (*P* = 6.1 × 10^−8^) but was only nominally significant among women (*P* = 0.01).

**Table 5:** Interaction terms between gout-associated SNPs and all alcohol intake (units/week) for gout. Significant interactions highlighted in bold

**Locus**	**SNP**	**Gout risk allele**	**All participants (*N* = 458,367)**				**Men (*N* = 209,493)**				**Women (*N* = 248,874)**			
			**Obs, %**	**IT (OR)**	**95% CI**	***P*-value**	**Obs, %**	**IT (OR)**	**95% CI**	***P*-value**	**Obs, %**	**IT (OR)**	**95% CI**	***P*-value**
*GCKR*	rs1260326	T	76.4%	1.000	0.998, 1.002	0.80	80.0%	1.000	0.998, 1.002	0.78	73.3%	0.996	0.984, 1.008	0.51
***ADH1B***	**rs1229984**	**T**	**76.4%**	**1.012**	**1.008, 1.016**	**8.15** × **10**^−**9**^	**80.0%**	**1.011**	**1.007, 1.016**	**6.07** × **10**^−**8**^	73.3%	1.027	1.006, 1.048	0.01
*MLXIPL*	rs6460047	T	76.0%	0.999	0.994, 1.003	0.60	79.6%	0.999	0.994, 1.004	0.68	72.9%	0.983	0.959, 1.008	0.18
*NFAT5*	rs113441031	T	75.8%	0.998	0.997, 1.000	0.08	79.5%	0.998	0.997, 1.000	0.09	72.8%	1.000	0.987, 1.013	0.99

*Abbreviations*: *SNP* Single-nucleotide polymorphism, *Obs* Observations (%), *IT* Interaction term for SNP (≥one urate-raising allele vs. none) × all alcohol intake (units/week), *OR* Odds ratio, *95% CI* 95% confidence interval

Adjusted for age, gender (all participants only), Townsend deprivation index, BMI, diuretics use, eGFR, high cholesterol, hypertension, cardiac problem, peripheral vascular disease, stroke, diabetes mellitus, smoking status, meat intake, fish intake, coffee intake, tea intake, fruit intake, vegetable intake, bread intake, cereal intake, and cheese intake

Bonferroni-corrected experiment-wide significance for each analysis: *P* < 0.01

Between the individual alcoholic beverage types, beer intake demonstrated the most significant interaction with *ADH1B* rs1229984 for association with gout among men, while wine intake had the most significant interaction among women (Additional file 1: Table S8). There was no evidence for interaction with spirits intake for association with gout.

### Genotype-stratified association analysis for serum urate level

Non-additive effects between alcohol consumption and rs1229984 at the *ADH1B* locus and rs6460047 at the *MLXIPL* locus were observed for association with serum urate level. Among the subgroup of all participants who did not consume any alcohol, the presence of the urate-raising T allele at *ADH1B* rs1229984 was not associated with serum urate level (*P* = 0.46) (Table 6; and Additional file 1: Table S10). However, among those who did consume alcohol, the T-positive genotype was associated with a 6.2 μmol/L increase in serum urate level compared to the T-negative genotype (*P*_difference_ = 1.5 × 10^−47^). In men and women separately, the T allele was again associated with increased serum urate level among alcohol consumers (*P* = 3.7 × 10^−28^ and *P* = 6.6 × 10^−21^, respectively) but not non-consumers (*P* = 0.81 and *P* = 0.75, respectively).

**Table 6:** Genotype-stratified association analysis of *ADH1B* or *MLXIPL* with alcohol intake for serum urate level (μmol/L)

**Group**	***Locus* SNP**	**Urate-raising allele**	***N***	**No alcohol intake**		**Any alcohol intake**		
				***β* (SE)**	***P*-value**	***β* (SE)**	***P*-value**	***P***_**difference**_
All participants				*N* = 26,472		*N* = 382,105		
	*ADH1B*	T−	*N* = 387,563	1	-	10.35 (0.38)	3.06 × 10^−160^	
	rs1229984	T+	*N* = 21,014	1.07 (1.46)	0.46	16.53 (0.56)	1.42 × 10^−192^	1.49 × 10^−47^
				*N* = 26,328		*N* = 380,042		
	*MLXIPL*	T−	*N* = 17,344	1	-	8.58 (1.78)	1.38 × 10^−6^	
	rs6460047	T+	*N* = 389,026	2.67 (1.75)	0.13	13.38 (1.72)	7.30 × 10^−15^	3.99 × 10^−25^
Men				*N* = 8937		*N* = 174,876		
	*ADH1B*	T−	*N* = 174,229	1	-	16.32 (0.70)	7.26 × 10^−121^	
	rs1229984	T+	*N* = 9584	0.68 (2.83)	0.81	23.64 (0.94)	1.06 × 10^−138^	3.65 × 10^−28^
				*N* = 8889		*N* = 173,949		
	*MLXIPL*	T−	*N* = 7699	1	-	10.99 (3.27)	7.91 × 10^−4^	
	rs6460047	T+	*N* = 175,139	−0.16 (3.26)	0.96	16.72 (3.20)	1.70 × 10^−7^	1.06 × 10^−14^
Women				*N* = 17,535		*N* = 207,229		
	*ADH1B*	T−	*N* = 213,334	1	-	8.46 (0.44)	1.28 × 10^−82^	
	rs1229984	T+	*N* = 11,430	0.50 (1.61)	0.75	13.50 (0.68)	8.96 × 10^−89^	6.59 × 10^−21^
				*N* = 17,439		*N* = 206,093		
	*MLXIPL*	T−	*N* = 9645	1	-	8.66 (2.02)	1.80 × 10^−5^	
	rs6460047	T+	*N* = 213,887	3.89 (1.98)	0.05	12.62 (1.94)	8.43 × 10^−11^	7.04 × 10^−12^

*Abbreviations*: *SNP* Single-nucleotide polymorphism, *OR* Odds ratio of hyperuricaemia compared to reference group (no urate-raising allele × no alcohol intake), *95% CI* 95% confidence interval, *P*_*difference*_ *P*-value for odds ratio of hyperuricaemia for presence vs. absence of urate-raising allele in alcohol-exposed (any alcohol intake) subgroup

Adjusted for age, gender (all participants only), Townsend deprivation index, BMI, diuretics use, eGFR, high cholesterol, hypertension, cardiac problem, peripheral vascular disease, stroke, diabetes mellitus, smoking status, meat intake, fish intake, coffee intake, tea intake, fruit intake, vegetable intake, bread intake, cereal intake, and cheese intake

Bonferroni-corrected experiment-wide significance for each analysis: *P* < 0.03

Similarly, the urate-raising T allele at *MLXIPL* rs6460047 did not associate with serum urate level among all non-consumers (*P* = 0.13) but was associated with a 4.8 µmol/L increase in serum urate level among all consumers (*P*_difference_ = 4.0 × 10^−25^). After stratifying by gender, the serum urate-increasing effect of the T allele at *MLXIPL* rs6460047 was again only observed among alcohol consumers in both men (*P*_difference_ = 1.1 × 10^−14^) and women (*P*_difference_ = 7.0 × 10^−12^).

Between the individual alcoholic beverage types, the increase in serum urate level in participants who consumed each alcoholic beverage type and had the urate-raising allele compared to the reference group (no intake of alcoholic beverage type and no urate-raising allele) was largest for beer intake at both *ADH1B* rs1229984 and *MLXIPL* rs6460047 among all participants (Additional file 1: Table S11). After stratifying by gender, this remained the case among men but wine intake demonstrated the largest increase in serum urate level among women.

### Genotype-stratified association analysis for hyperuricaemia

Among the subgroup of all participants who did not consume alcohol, the presence of the urate-raising T allele at *ADH1B* rs1229984 was not associated with hyperuricaemia (*P* = 0.39) (Table 7; and Additional file 1: Table S12). In contrast, there was significant association with hyperuricaemia among alcohol consumers; the odds ratio of hyperuricaemia for the T-positive genotype compared to the T-negative genotype in the alcohol-exposed subgroup was 1.3 (*P*_difference_ = 4.1 × 10^−20^). The same relationship was seen in the gender-stratified analysis, with the T-positive genotype conferring increased association with hyperuricaemia among drinkers in both men (*P*_difference_ = 2.2 × 10^−17^) and women (*P*_difference_ = 0.001).

**Table 7:** Genotype-stratified association analysis of *ADH1B* and alcohol intake for hyperuricaemia

**Group**	***Locus* SNP**	**Urate-raising allele**	***N***	**No alcohol intake**		**Any alcohol intake**		
				**OR (95% CI)**	***P*-value**	**OR (95% CI)**	***P*-value**	***P***_**difference**_
All participants				*N* = 26,472		*N* = 382,105		
	*ADH1B*	T−	*N* = 387,563	1	-	1.48 (1.40, 1.56)	1.65 × 10^−43^	
	rs1229984	T+	*N* = 21,014	1.10 (0.89, 1.35)	0.39	1.86 (1.73, 2.00)	1.47 × 10^−63^	4.09 × 10^−20^
Men				*N* = 8937		*N* = 174,876		
	*ADH1B*	T−	*N* = 174,229	1	-	1.67 (1.56, 1.79)	3.39 × 10^−50^	
	rs1229984	T+	*N* = 9584	1.08 (0.83, 1.41)	0.55	2.11 (1.94, 2.29)	5.07 × 10^−67^	2.20 × 10^−17^
Women				*N* = 17,535		*N* = 207,229		
	*ADH1B*	T−	*N* = 213,334	1	-	1.29 (1.17, 1.42)	1.45 × 10^−7^	
	rs1229984	T+	*N* = 11,430	1.06 (0.75, 1.49)	0.76	1.59 (1.37, 1.85)	2.57 × 10^−9^	0.001

*Abbreviations*: *SNP* Single-nucleotide polymorphism, *OR* Odds ratio of hyperuricaemia compared to reference group (no urate-raising allele × no alcohol intake), *95% CI* 95% confidence interval, *P*_*difference*_ *P*-value for odds ratio of hyperuricaemia for presence vs. absence of urate-raising allele in alcohol-exposed (any alcohol intake) subgroup

Adjusted for age, gender (all participants only), Townsend deprivation index, BMI, diuretics use, eGFR, high cholesterol, hypertension, cardiac problem, peripheral vascular disease, stroke, diabetes mellitus, smoking status, meat intake, fish intake, coffee intake, tea intake, fruit intake, vegetable intake, bread intake, cereal intake, and cheese intake

Between the individual alcoholic beverage types, the odds ratio of hyperuricaemia in participants who consumed each alcoholic beverage type and had the urate-raising allele compared to the reference group (no intake of alcoholic beverage type and no urate-raising allele) was observed to be largest for beer intake among all participants (Additional file 1: Table S13). After stratifying by gender, this remained the case among men but there was no significant difference between the alcoholic beverage types among women.

### Genotype-stratified association analysis for gout

Non-additive effects with alcohol intake were also observed at *ADH1B* rs1229984 for gout (Table 8 and Additional file 1: Table S14). Among all participants, the presence of the T allele was not associated with gout among non-consumers (*P* = 0.84). However, in the alcohol-exposed subgroup, the associated risk of gout was 1.8 times greater for the T-positive genotype compared to the T-negative genotype (*P*_difference_ = 3.4 × 10^−36^). A similar effect was also seen among men and women separately (*P*_difference_ = 1.1 × 10^−33^ and *P*_difference_ = 0.001, respectively).

**Table 8:** Genotype-stratified association analysis of *ADH1B* and alcohol intake for gout

**Group**	***Locus* SNP**	**Gout risk allele**	***N***	**No alcohol intake**		**Any alcohol intake**		
				**OR (95% CI)**	***P*-value**	**OR (95% CI)**	***P*-value**	***P***_**difference**_
All participants				*N* = 26,738		*N* = 387,034		
	*ADH1B*	T−	*N* = 392,329	1	-	1.39 (1.22, 1.58)	6.88 × 10^−7^	
	rs1229984	T+	*N* = 21,443	0.95 (0.56, 1.59)	0.84	2.53 (2.17, 2.96)	7.17 × 10^−32^	3.42 × 10^−36^
Men				*N* = 9114		*N* = 179,232		
	*ADH1B*	T−	*N* = 178,369	1	-	1.65 (1.42, 1.91)	6.09 × 10^−11^	
	rs1229984	T+	*N* = 9977	0.90 (0.48, 1.69)	0.75	3.01 (2.53, 3.58)	3.81 × 10^−35^	1.06 × 10^−33^
Women				*N* = 17,624		*N* = 207,802		
	*ADH1B*	T−	*N* = 213,960	1	-	0.78 (0.60, 1.01)	0.06	
	rs1229984	T+	*N* = 11,466	1.02 (0.41, 2.55)	0.97	1.42 (0.93, 2.17)	0.10	0.001

*Abbreviations*: *SNP* Single-nucleotide polymorphism, *OR* Odds ratio of gout compared to reference group (no gout risk allele × no alcohol intake), *95% CI* 95% confidence interval, *P*_*difference*_ *P*-value for odds ratio of gout for presence vs. absence of gout risk allele in alcohol-exposed (any alcohol intake) subgroup

Adjusted for age, gender (all participants only), Townsend deprivation index, BMI, diuretics use, eGFR, high cholesterol, hypertension, cardiac problem, peripheral vascular disease, stroke, diabetes mellitus, smoking status, meat intake, fish intake, coffee intake, tea intake, fruit intake, vegetable intake, bread intake, cereal intake, and cheese intake

After stratifying by alcoholic beverage type, the odds ratio of gout in participants who consumed each alcoholic beverage type and had the gout risk-increasing allele compared to the reference group (no intake of alcoholic beverage type and no gout risk-increasing allele) was largest for beer intake among all participants (Additional file 1: Table S15). This remained the case among men but there was no significant difference between the alcoholic beverage types among women.

## Discussion

This study investigated the interactions between urate and gout-associated SNPs and alcohol consumption for association with serum urate level, hyperuricaemia, and gout using cross-sectional data from more than 400,000 individuals of European ethnicity in the UK Biobank. Our findings provide further evidence for the association of alcohol intake with elevated serum urate level and gout risk. We observed significant interactions with alcohol consumption at rs1229984 in the *ADH1B* locus and rs6460047 in the *MLXIPL* locus for association with serum urate level, and at *ADH1B* rs1229984 only for association with hyperuricaemia and gout. In the genotype-stratified analysis, these loci were associated with serum urate level, hyperuricaemia, or gout among alcohol consumers but not non-consumers.

We observed that alcohol consumption was associated with increased serum urate level in both genders, although the effect was larger among men than women. This differs from other studies which found no significant difference between sexes [2, 20] or significant effects in males but an absence of effect among females [21, 22]. Consistent with previous studies, beer intake was associated with the largest increase in serum urate level and gout, followed by spirits [2–4, 20, 23, 24]. While moderate wine intake did not associate with serum urate or gout in several earlier studies [2–4, 20], we demonstrated significant association of wine consumption with increased serum urate level among all groups and with gout among men. Compared to these earlier studies, the present study had a much larger sample size with a higher proportion of wine consumers, more extensive wine intake data, and more comprehensive adjustment for covariables. The serum urate level and hyperuricaemia risk-increasing effect of wine consumption has more recently been demonstrated among a large cohort of Japanese participants, with each unit increase in daily wine intake associating with a 0.10 mg/dL increase in serum urate level among both males and females (95% CI 0.07–0.013), a 1.18 times greater risk of hyperuricaemia among males (95% CI 1.11–1.25), and a 1.22 times greater risk of hyperuricaemia among females (95% CI 1.10–1.34) [24]. The present study extends these findings to a European population. Although no association between wine intake and gout was observed for women in the present study, this may be due to the very small proportion of gout among women in the UK Biobank cohort (0.2% of women).

*ADH1B* encodes an alcohol dehydrogenase involved in the metabolism of alcohol into acetaldehyde. The fast-metabolising variant of *ADH1B* rs1229984 is associated with increased serum urate and gout among Japanese male consumers of alcohol [12, 13]. We similarly demonstrated this association among alcohol consumers and are the first to extend these findings to a European population and to women. The fast-metabolising variant of *ADH1B* is known to cause adverse reactions to alcohol consumption due to the build-up of acetaldehyde and is typically associated with reduced alcohol intake [25, 26]. However, Yokoyama and colleagues [12] proposed that among individuals who are heavy alcohol consumers despite having this variant, faster metabolism of alcohol leads to increased serum urate through the elevation of blood lactate levels [27], increased ATP degradation and purine content [28, 29], and induction of *CYP2E1* activity [30]. This suggests that the association of *ADH1B* with serum urate level and gout may occur through the modulation of alcohol metabolism rate rather than downstream effects on alcohol intake behaviour. However, other studies observed that the association of *ADH1B* with hyperuricaemia and gout remained significant after adjusting for alcohol consumption [12, 13], suggesting that *ADH1B* may also associate with serum urate and gout among alcohol consumers through other pathways.

We identified an additional novel interaction between alcohol consumption and rs6460047 at the *MLXIPL* locus for association with serum urate level among Europeans. Rs6460047 lies in the intergenic region between *MLXIPL* and *VPS37D*, with *MLXIPL* being the nearest gene. The serum urate signal of association at this locus has previously been linked to the expression of *MLXIPL* using Genotype and Tissue Expression data [6, 31]. The serum urate-increasing allele at this locus is associated with lowered expression of *MLXIPL* in multiple tissues. *MLXIPL* encodes for ChREBP (carbohydrate-responsive element-binding protein), a transcription factor that promotes the transcription of glycolysis and lipogenesis genes in metabolic tissues including the liver. In the presence of alcohol, ChREBP is post-translationally modified, activated, and translocated to the nucleus and functions as a transcriptional regulator of lipogenesis [32, 33]. In mice, ChREBP knockout leads to higher plasma urate levels [34]. Although it is difficult to ascertain the mechanism of action from these genetic data, it is possible that the serum urate-raising allele that lowers *MLXIPL* expression leads to reduced ChREBP transcriptional activity, with exacerbation of this effect in the presence of alcohol.

Between the individual alcoholic beverage types, beer intake was observed to have the most significant interaction with *ADH1B* rs1229984 for association with serum urate level, hyperuricaemia, and gout and with *MLXIPL* rs6460047 for association with serum urate level among all participants. The genotype-stratified association analysis demonstrated the largest increase in serum urate level or risk of hyperuricaemia or gout among participants who had the urate-increasing or gout risk allele and consumed beer, as opposed to spirits or wine. However, stratification by gender revealed that while this remained the case among men, wine intake had the most significant interaction with *ADH1B* for association with serum urate level, hyperuricaemia, and gout among women and demonstrated the largest increase in serum urate level in women who both had the urate-increasing allele and consumed wine. This likely reflects the much higher consumption of wine compared to beer among women in the study (mean of 11.16 units/week of wine compared to 1.24 units/week of beer). While there was no significant difference between the alcoholic beverage types in the genotype-stratified association analysis for association with hyperuricaemia and gout among women, this is likely to be driven by the much lower proportion of women with hyperuricaemia or gout compared to men in the study and therefore reduced statistical power. This suggests that between the individual alcoholic beverage types, beer intake is the greatest contributor to the genetic relationships observed among men while wine intake is the greatest contributor among women.

This study has some limitations. The analyses were restricted to individuals of European ethnicity between the ages of 40 and 69 years, limiting the generalisability of the findings. The frequency of the *ADH1B* rs1229984 variant is relatively low among Europeans, especially compared to East Asian populations (minor allele frequency 0.03 and 0.70, respectively) [35], which reduces the statistical power of the analyses. The single baseline serum urate measurement may not accurately represent the life-long urate level of each participant. The use of self-reported alcohol consumption data may have influenced the observed effect sizes to some degree due to underreporting as the result of recall and social desirability biases [36]. Additionally, around 15% of all participants were missing data for one or more alcohol consumption phenotypes. Given that only a small proportion of participants did not consume alcohol, the absence of genetic associations with serum urate level, hyperuricaemia, and gout in non-consumers may be due to reduced power among this subgroup in the genotype-stratified association analysis. Finally, due to the cross-sectional nature of the study, we cannot rule out the potential influence of collider bias or reverse causation in the gout analyses [37].

## Conclusion

In summary, this study has identified novel interactions with alcohol consumption at *ADH1B* and *MLXIPL* for association with serum urate level and at *ADH1B* for association with hyperuricaemia and gout among individuals of European ethnicity. The effects at these loci were only observed among alcohol consumers. The association of *ADH1B* with serum urate level, hyperuricaemia, and gout may occur through the modulation of alcohol metabolism rate in alcohol consumers, while the association of *MLXIPL* with serum urate level may occur through enhanced downregulation of ChREBP transcriptional activity among alcohol consumers.

### Supplementary Information

**Additional file 1:** **Supplementary Methods.** **Table S1.** Alcohol consumption GWAS included in study and source of datasets. **Table S2.** Gender-stratified baseline demographic and clinical characteristics of all eligible study participants. **Table S3.** Association of alcohol intake with serum urate level, hyperuricaemia, and gout using other models of adjustment for covariables. **Table S4.** Details from relevant GWAS of lead SNPs at genome-wide significance included in interaction analysis. **Table S5.** Interaction terms between urate-associated SNPs and all alcohol intake (units/week) for serum urate level (μmol/L) using other models of adjustment for covariables. **Table S6.** Interaction terms between urate-associated SNPs and all alcohol intake (units/week) for hyperuricaemia using other models of adjustment for covariables.**Table S7.** Interaction terms between *ADH1B* or *MLXIPL* and beer, spirits, and wine intake as continuous variables (units/week) for serum urate level (μmol/L). **Table S8.** Interaction terms between *ADH1B* and beer, spirits, and wine intake as continuous variables (units/week) for hyperuricaemia and gout. **Table S9.** Interaction terms between gout-associated SNPs and all alcohol intake (units/week) for gout using other models of adjustment for covariables. **Table 10.** Genotype-stratified association analysis of *ADH1B* or *MLXIPL* and binarized alcohol intake for serum urate level using other models of adjustment for covariables. **Table S11.** Genotype-stratified association analysis of *ADH1B* or *MLXIPL* and binarized beer, spirits, and wine intake for serum urate level (μmol/L). **Table S12.** Genotype-stratified association analysis of *ADH1B* and binarized alcohol intake for hyperuricaemia using other models of adjustment for covariables. **Table S13.** Genotype-stratified association analysis of *ADH1B* and binarized beer, spirits, and wine intake for hyperuricaemia. **Table S14.** Genotype-stratified association analysis of *ADH1B* and binarized alcohol intake for gout using other models of adjustment for covariables. **Table S15.** Genotype-stratified association analysis of *ADH1B* and binarized beer, spirits, and wine intake for gout. **Figure S1.** Distribution of all alcohol intake (units/week) data among all participants. **Figure S2.** LocusZoom plots within UK Biobank for five serum urate loci included in interaction analysis. **Figure S3.** LocusZoom plots within UK Biobank for four gout loci included in interaction analysis.

## Data Availability

Data are available through the UK Biobank resource.

## Abbreviations

ABCG2: ATP-binding cassette subfamily G member 2
ADH1B: Alcohol dehydrogenase 1B
ALDH2: Aldehyde dehydrogenase 2
A1CF: APOBEC1 complementation factor
BMI: Body mass index
ChREBP: Carbohydrate-responsive element-binding protein
CI: Confidence interval
CKD-EPI: Chronic Kidney Disease Epidemiology Collaboration
CYP2E1: Cytochrome P450 family 2 subfamily E member 1
eGFR: Estimated glomerular filtration rate
GCKR: Glucokinase regulatory protein
GWAS: Genome-wide association study
LRP2: Low-density lipoprotein receptor-related protein
MLXIPL: MLX interacting protein-like
OR: Odds ratio
SE: Standard error
SNP: Single-nucleotide polymorphism
UK: United Kingdom
