# Supplementary material for: Interaction of genetic variation at ADH1B and MLXIPL with alcohol consumption for elevated serum urate level and gout among people of European ethnicity
Source: Arthritis Res Ther. 2024 Feb 8;26:45. doi: 10.1186/s13075-024-03279-9 (PMC10851571; doi:10.1186/s13075-024-03279-9)
Supplement: Supplementary file 1 — Additional file 1: Supplementary Methods. Table S1. Alcohol consumption GWAS included in study and source of datasets. Table S2. Gender-stratified baseline demographic and clinical characteristics of all eligible study participants. Table S3. Association of alcohol intake with serum urate level, hyperuricaemia, and gout using other models of adjustment for covariables. Table S4. Details from relevant GWAS of lead SNPs at genome-wide significance included in interaction analysis. Table S5. Interaction terms between urate-associated SNPs and all alcohol intake (units/week) for serum urate level (μmol/L) using other models of adjustment for covariables. Table S6. Interaction terms between urate-associated SNPs and all alcohol intake (units/week) for hyperuricaemia using other models of adjustment for covariables.Table S7. Interaction terms between ADH1B or MLXIPL and beer, spirits, and wine intake as continuous variables (units/week) for serum urate level (μmol/L). Table S8. Interaction terms between ADH1B and beer, spirits, and wine intake as continuous variables (units/week) for hyperuricaemia and gout. Table S9. Interaction terms between gout-associated SNPs and all alcohol intake (units/week) for gout using other models of adjustment for covariables. Table 10. Genotype-stratified association analysis of ADH1B or MLXIPL and binarized alcohol intake for serum urate level using other models of adjustment for covariables. Table S11. Genotype-stratified association analysis of ADH1B or MLXIPL and binarized beer, spirits, and wine intake for serum urate level (μmol/L). Table S12. Genotype-stratified association analysis of ADH1B and binarized alcohol intake for hyperuricaemia using other models of adjustment for covariables. Table S13. Genotype-stratified association analysis of ADH1B and binarized beer, spirits, and wine intake for hyperuricaemia. Table S14. Genotype-stratified association analysis of ADH1B and binarized alcohol intake for gout using other models of adj [file 13075_2024_3279_MOESM1_ESM.docx]

Supplementary Materials for

**Interaction of Genetic Variation at *ADH1B* and *MLXIPL* with Alcohol Consumption for Elevated Serum Urate Level and Gout among People of European Ethnicity**

Min H Chuah, Megan P Leask, Ruth K Topless, Gregory D Gamble, Nicholas A Sumpter, Lisa K Stamp,
Tony R Merriman, Nicola Dalbeth

**This file includes:**

Supplementary Methods

Supplementary Tables

Table S1. Alcohol consumption GWAS included in study and source of datasets.

Table S2. Gender-stratified baseline demographic and clinical characteristics of all eligible study participants.

Table S3. Association of alcohol intake with serum urate level, hyperuricaemia, and gout using other models of adjustment for covariables.

Table S4. Details from relevant GWAS of lead SNPs at genome-wide significance included in interaction analysis.

Table S5. Interaction terms between urate-associated SNPs and all alcohol intake (units/week) for serum urate level (μmol/L) using other models of adjustment for covariables.

Table S6. Interaction terms between urate-associated SNPs and all alcohol intake (units/week) for hyperuricaemia using other models of adjustment for covariables.

Table S7. Interaction terms between *ADH1B* or *MLXIPL* and beer, spirits, and wine intake as continuous variables (units/week) for serum urate level (μmol/L).

Table S8. Interaction terms between *ADH1B* and beer, spirits, and wine intake as continuous variables (units/week) for hyperuricaemia and gout.

Table S9. Interaction terms between gout-associated SNPs and all alcohol intake (units/week) for gout using other models of adjustment for covariables.

Table 10. Genotype-stratified association analysis of *ADH1B* or *MLXIPL* and binarized alcohol intake for serum urate level using other models of adjustment for covariables.

Table S11. Genotype-stratified association analysis of *ADH1B* or *MLXIPL* and binarized beer, spirits, and wine intake for serum urate level (μmol/L).

Table S12. Genotype-stratified association analysis of *ADH1B* and binarized alcohol intake for hyperuricaemia using other models of adjustment for covariables.

Table S13. Genotype-stratified association analysis of *ADH1B* and binarized beer, spirits, and wine intake for hyperuricaemia.

Table S14. Genotype-stratified association analysis of *ADH1B* and binarized alcohol intake for gout using other models of adjustment for covariables.

Table S15. Genotype-stratified association analysis of *ADH1B* and binarized beer, spirits, and wine intake for gout.

Supplementary Figures

Figure S1. Distribution of all alcohol intake (units/week) data among all participants.

Figure S2. LocusZoom plots within UK Biobank for five serum urate loci included in interaction analysis.

Figure S3. LocusZoom plots within UK Biobank for four gout loci included in interaction analysis.

**Supplementary Methods**

*Calculation and standardisation of weekly alcohol intake data*

Self-reported alcohol consumption data were obtained through the UK Biobank baseline Touchscreen Questionnaire. All participants who indicated that they consume alcohol further quantified their weekly or monthly intake of beer/cider (pints), spirits/liqueur (measures), red wine (glasses), white wine/champagne (glasses), fortified wine (glasses), and other alcoholic beverages (glasses). Where participants gave a response of greater than zero for weekly or monthly intake of at least one alcoholic beverage type, missing responses for the other beverage types were assumed to indicate no consumption. Participants who answered “Don’t know” or “Prefer not to answer” for a particular measure of alcohol intake were coded as missing data. All data for monthly alcohol intake were converted into weekly intake using the following formula: weekly alcohol intake = monthly alcohol intake ÷ ^52^/_12_.

The standardised number of UK alcohol units assigned to each alcoholic drink in the study were as follows: beer/cider (pint) = **2.5** units; spirits/liquor (measure) = **1** unit; red wine (glass) = **2** units; white wine (glass) = **2** units; fortified wine (glass) = **2** units; other alcohol (glass) = **1.5** units [1,2]. To calculate the weekly units of alcohol consumed, the units of alcohol assigned to each alcoholic beverage type were multiplied by the corresponding number of drinks consumed per week.

All wine intake was derived from the sum of units per week intake of red wine, white wine, and fortified wine. All alcohol intake was calculated from the sum of units per week intake of all the alcoholic beverage types. Any missing data for the individual alcoholic beverage types included in calculating the above summative phenotypes resulted in coding of the data for these phenotypes as missing. Values greater than zero were classified as any alcohol intake.

References

Howe LJ, Lawson DJ, Davies NM, St Pourcain B, Lewis SJ, Davey Smith G, Hemani G. Genetic evidence for assortative mating on alcohol consumption in the UK Biobank. *Nature Communications*. 2019;10(1):5039.

UK National Health Service. Alcohol units [Internet]. nhs.uk; [cited 2021 May 11]. Available from: <https://www.nhs.uk/live-well/alcohol-support/calculating-alcohol-units/>

*Calculation of other dietary intake variables*

Relevant dietary intake data included meat (processed meat, beef, lamb/mutton, pork, poultry), fish (oily, non-oily), coffee, tea, fruit (fresh, dried), vegetables (raw, cooked), bread, cereal, and cheese.

The UK Biobank baseline Touchscreen Questionnaire collected data on frequency of meat, fish, and cheese intake as binned responses. In the present study, these were recoded to represent the midpoint of each range. All dietary data collected as daily intake were converted into weekly intake values by multiplying by 7. Weekly frequency of consumption was assumed to be equivalent to the number of servings per week.

A response of “Don’t know” or “Prefer not to answer” for intake of an individual food item was coded as missing data. Total intake of a particular food group was calculated by summing the intake of the individual food items in each group (listed in brackets above). Any missing data for the individual food items resulted in total intake of the food group being coded as missing.

**Table S1. Alcohol consumption GWAS included in study and source of datasets.**

| **Study; *journal*** | **Dataset name** | **Dataset source** | **Additional notes** |
| --- | --- | --- | --- |
| Schumann et al. (2016) [1]; *Proceedings of the National Academy of Sciences of the United States of America* | A Genome-Wide Association Meta-Analysis of Alcohol Drinking as a Continuous Trait (accession: pha004513.1) | dbGaP; CHARGE (Cohorts for Heart and Aging Research in Genomic Epidemiology) Consortium Summary Results from Genomic Studies (accession: [phs000930](http://www.ncbi.nlm.nih.gov/projects/gap/cgi-bin/study.cgi?study_id=phs000930).v9.p1), [https://www.ncbi.nlm.nih.gov/ projects/gap/cgi-bin/study.cgi? study_id=phs000930.v9.p1](https://www.ncbi.nlm.nih.gov/projects/gap/cgi-bin/study.cgi?study_id=phs000930.v9.p1) | Grams per day; 85,510 Europeans |
|  | A Genome-Wide Association Meta-Analysis of Alcohol Drinking as a Dichotomous Trait (accession: pha004516.1) |  | Heavy vs. light or no drinking; 81,925 Europeans |
|  | *Table 1.* Associations of SNPs with alcohol intake (log grams per day) in the GWAS analysis | Publication (*Table 1*) | Combined analysis (GWAS and replication) |
| Jorgenson et al. (2017) [2]; *Molecular Psychiatry* | *Table 2.* Top genome-wide associations with alcohol consumption in each individual race/ethnicity group and in the trans-ethnic meta-analysis | Publication (*Table 2*) | Drinker status (dichotomous), drinks per week |
| Clarke et al. (2017) [3]; *Molecular Psychiatry* | *Table 1.* Fourteen loci reaching genome-wide significance for association with alcohol consumption in UKB after performing clump-based pruning | Publication (*Table 1*) | Units per week |
|  | *Supplementary Table 3.* Loci reaching genome-wide significance for association with alcohol consumption in UKB in males and females only | Publication (supplementary material) |  |
| Liu et al. (2019) [4]; *Nature Genetics* | [Drinks per Week Summary Statistics](https://conservancy.umn.edu/bitstream/handle/11299/201564/DrinksPerWeek.txt.gz?sequence=32&isAllowed=y) | University of Minnesota Digital Conservancy (UDC), https://conservancy.umn. edu/handle/11299/201564 | Drinks per week; full study includes 941,280 Europeans, but data excludes 23andMe cohort due to legal constraints |
|  | *Supplementary Table 5.* Genome-Wide Significant Conditionally Independent Association Results for Drinks per Week | Publication (supplementary material) | Drinks per week; independent associated variants |
| Gelernter et al. (2019) [5]; *Biological Psychiatry* | MaxAlc.EUR.MVP.BiolPsych2019 (accession: pha004849.1) | dbGaP; Veterans Administration (VA) Million Veteran Program (MVP) Summary Results from Omics Studies (accession: [phs001672.v4.p1](https://www.ncbi.nlm.nih.gov/projects/gap/cgi-bin/study.cgi?study_id=phs001672.v4.p1)), [https://www.ncbi.nlm.nih.gov/ projects/gap/cgi-bin/study. cgi?study_id=phs001672.v1.p1](https://www.ncbi.nlm.nih.gov/%20projects/gap/cgi-bin/study.%20cgi?study_id=phs001672.v1.p1) | Maximum habitual alcohol intake; 126,936 Europeans |
| Thompson et al. (2020) [6]; *Science Advances* | Summary_Stats_Thompson.txt | Email request (corresponding author: Dr A. Thompson) | Heavy drinker; 173,216 Europeans |
| Cole et al. (2020) [7]; *Nature Communications* | Supplementary Dataset 4 | Publication (supplementary information) | Top dietary habit |

References

1. Schumann G, Liu C, O’Reilly P, Gao H, Song P, Xu B, et al. KLB is associated with alcohol drinking, and its gene product β-Klotho is necessary for FGF21 regulation of alcohol preference. *Proc Natl Acad Sci U S A*. 2016;113(50):14372-7.
2. Jorgenson E, Thai KK, Hoffmann TJ, Sakoda LC, Kvale MN, Banda Y, et al. Genetic contributors to variation in alcohol consumption vary by race/ethnicity in a large multi-ethnic genome-wide association study. *Mol Psychiatry*. 2017;22(9):1359-67.
3. Clarke TK, Adams MJ, Davies G, Howard DM, Hall LS, Padmanabhan S, et al. Genome-wide association study of alcohol consumption and genetic overlap with other health-related traits in UK Biobank (N = 112 117). *Mol Psychiatry*. 2017;22(10):1376-84.
4. Liu M, Jiang Y, Wedow R, Li Y, Brazel DM, Chen F, et al. Association studies of up to 1.2 million individuals yield new insights into the genetic etiology of tobacco and alcohol use. *Nat Genet*. 2019;51(2):237-44.
5. Gelernter J, Sun N, Polimanti R, Pietrzak R, Levey DF, Lu Q, et al. Genome-wide association study of maximum habitual alcohol intake in >140,000 U.S. European and African American veterans yields novel risk loci. *Biol Psychiatry*. 2019;86(5):365-76.
6. Thompson A, Cook J, Choquet H, Jorgenson E, Yin J, Kinnunen T, et al. Functional validity, role, and implications of heavy alcohol consumption genetic loci. *Sci Adv*. 2020;6(3):eaay5034.
7. Cole JB, Florez JC, Hirschhorn JN. Comprehensive genomic analysis of dietary habits in UK Biobank identifies hundreds of genetic associations. *Nat Commun*. 2020;11(1):1467.

**Table S2. Gender-stratified baseline demographic and clinical characteristics of all eligible study participants.** Data shown as mean (standard deviation) unless indicated otherwise.

|  | **All eligible men** | **All eligible women** |
| --- | --- | --- |
| *N (%)* | 209,524 | 248,881 |
| Age (years) | 56.99 (8.11) | 56.58 (7.94) |
| Townsend deprivation index | -1.42 (3.05) | -1.49 (2.94) |
| Gout, *N* (%) | 6,664 (3.2%) | 559 (0.2%) |
| Urate-lowering therapy use, *N* (%) | 4,840 (2.3%) | 404 (0.2%) |
| Serum urate level (μmol/L) | 354.67 (71.68) | 270.65 (66.01) |
| Hyperuricaemia (≥410 μmol/L), *N* (%) | 41,218 (20.6%) | 7,345 (3.1%) |
| Serum creatinine level (μmol/L) | 81.59 (18.13) | 64.35 (13.31) |
| eGFR (mL/min/1.73m^2^) | 90.38 (13.27) | 90.65 (13.25) |
| Diuretics, *N* (%) | 16,705 (8.0%) | 21,424 (8.6%) |
| Body mass index (kg/m^2^) | 27.85 (4.24) | 27.01 (5.14) |
| High cholesterol, *N* (%) | 31,950 (15.2%) | 24,251 (9.7%) |
| Hypertension, *N* (%) | 62,536 (29.8%) | 56,162 (22.6%) |
| Cardiac problem, *N* (%) | 755 (0.4%) | 711 (0.3%) |
| Peripheral vascular disease, *N* (%) | 339 (0.2%) | 526 (0.2%) |
| Stroke, *N* (%) | 3,639 (1.7%) | 2,423 (1.0%) |
| Diabetes, *N* (%) | 11,523 (5.5%) | 6,472 (2.6%) |
| Current smoker, *N* (%) | 25,517 (12.2%) | 22,228 (9.0%) |
| Previous smoker, *N* (%) | 82,124 (39.3%) | 80,469 (32.4%) |
| Never smoked, *N* (%) | 101,133 (48.4%) | 145,300 (58.6%) |
| Current alcohol drinker, *N* (%) | 198,727 (94.9%) | 228,525 (91.9%) |
| Previous alcohol drinker, *N* (%) | 6,933 (3.3%) | 9,011 (3.6%) |
| Never alcohol drinker, *N* (%) | 3,678 (1.8%) | 11,119 (4.5%) |
| All alcohol intake (units per week) | 25.74 (23.19) | 13.61 (13.81) |
| Beer intake (units per week) | 11.91 (16.76) | 1.24 (4.07) |
| Spirits intake (units per week) | 1.94 (5.95) | 1.18 (3.55) |
| All wine intake (units per week) | 11.92 (15.91) | 11.16 (12.73) |
| Total meat intake (servings per week) | 6.10 (2.80) | 5.05 (2.53) |
| Total fish intake (servings per week) | 2.24 (1.58) | 2.29 (1.56) |
| Coffee intake (cups per week) | 15.59 (15.66) | 13.52 (13.81) |
| Tea intake (cups per week) | 24.63 (21.24) | 23.91 (19.43) |
| Total fruit intake (pieces per week) | 19.07 (17.48) | 23.18 (17.29) |
| Total vegetable intake (tablespoons per week) | 32.30 (22.83) | 35.34 (21.71) |
| Bread intake (slices per week) | 15.34 (9.62) | 10.01 (6.79) |
| Cereal intake (bowls per week) | 4.53 (2.85) | 4.62 (2.72) |
| Cheese intake (servings per week) | 2.53 (1.76) | 2.33 (1.76) |

**Table S3. Association of alcohol intake with serum urate level, hyperuricaemia, and gout using other models of adjustment for covariables.**

| **Group** | **Alcohol intake (units/week)** | **Adjustment** | **Association with serum urate (μmol/L)** | | | **Association with hyperuricaemia** | | **Association with gout** | |
| --- | --- | --- | --- | --- | --- | --- | --- | --- | --- |
|  |  |  | **β (SE)** | **B** | ***P*-value** | **OR (95% CI)** | ***P*-value** | **OR (95% CI)** | ***P*-value** |
|  | | | *N* = 366,550 | | | *N* = 366,550 | | *N* = 389,924 | |
| All participants | All alcohol | Unadjusted | 0.49 (0.01) | 0.12 | < 1.00 x 10^-300^ | 1.023 (1.023, 1.024) | < 1.00 x 10^-300^ | 1.021 (1.021, 1.022) | < 1.00 x 10^-300^ |
|  |  | Age, gender, TDI | 0.48 (0.01) | 0.12 | < 1.00 x 10^-300^ | 1.013 (1.013, 1.013) | < 1.00 x 10^-300^ | 1.014 (1.013, 1.015) | 1.00 x 10^-248^ |
|  |  | Incl. comorbidities * | 0.55 (0.01) | 0.13 | < 1.00 x 10^-300^ | 1.017 (1.016, 1.017) | < 1.00 x 10^-300^ | 1.015 (1.014, 1.016) | 1.08 x 10^-246^ |
|  | Beer | Unadjusted | 1.98 (0.01) | 0.31 | < 1.00 x 10^-300^ | 1.038 (1.037, 1.039) | < 1.00 x 10^-300^ | 1.032 (1.031, 1.033) | < 1.00 x 10^-300^ |
|  |  | Age, gender, TDI | 0.78 (0.01) | 0.12 | < 1.00 x 10^-300^ | 1.019 (1.018, 1.020) | < 1.00 x 10^-300^ | 1.022 (1.021, 1.023) | < 1.00 x 10^-300^ |
|  |  | Incl. comorbidities * | 0.78 (0.01) | 0.12 | < 1.00 x 10^-300^ | 1.023 (1.022, 1.023) | < 1.00 x 10^-300^ | 1.024 (1.023, 1.025) | < 1.00 x 10^-300^ |
|  | Spirits | Unadjusted | 1.61 (0.03) | 0.10 | < 1.00 x 10^-300^ | 1.031 (1.030, 1.033) | 1.81 x 10^-275^ | 1.022 (1.020, 1.025) | 7.87 x 10^-65^ |
|  |  | Age, gender, TDI | 0.84 (0.02) | 0.05 | 6.44 x 10^-283^ | 1.019 (1.017, 1.021) | 5.87 x 10^-101^ | 1.013 (1.010, 1.016) | 2.36 x 10^-19^ |
|  |  | Incl. comorbidities * | 0.58 (0.02) | 0.03 | 7.70 x 10^-177^ | 1.016 (1.014, 1.018) | 3.65 x 10^-64^ | 1.009 (1.006, 1.012) | 6.54 x 10^-8^ |
|  | Wine | Unadjusted | 0.28 (0.01) | 0.05 | 2.84 x 10^-199^ | 1.006 (1.005, 1.007) | 1.57 x 10^-75^ | 1.002 (1.001, 1.004) | 0.01 |
|  |  | Age, gender, TDI | 0.22 (0.01) | 0.04 | 2.31 x 10^-172^ | 1.005 (1.004, 1.005) | 7.92 x 10^-47^ | 1.001 (0.999, 1.002) | 0.41 |
|  |  | Incl. comorbidities * | 0.34 (0.01) | 0.06 | < 1.00 x 10^-300^ | 1.008 (1.008, 1.009) | 1.59 x 10^-122^ | 1.003 (1.001, 1.004) | 2.19 x 10^-4^ |
|  | | | *N* = 174,074 | | | *N* = 174,074 | | *N* = 187,221 | |
| Men | All alcohol | Unadjusted | 0.53 (0.01) | 0.17 | < 1.00 x 10^-300^ | 1.013 (1.013, 1.014) | < 1.00 x 10^-300^ | 1.014 (1.013, 1.015) | 1.49 x 10^-238^ |
|  |  | Age and TDI | 0.54 (0.01) | 0.17 | < 1.00 x 10^-300^ | 1.013 (1.013, 1.014) | < 1.00 x 10^-300^ | 1.014 (1.014, 1.015) | 1.42 x 10^-250^ |
|  |  | Incl. comorbidities * | 0.57 (0.01) | 0.18 | < 1.00 x 10^-300^ | 1.016 (1.016, 1.017) | < 1.00 x 10^-300^ | 1.015 (1.014, 1.016) | 3.19 x 10^-243^ |
|  | Beer | Unadjusted | 0.74 (0.01) | 0.17 | < 1.00 x 10^-300^ | 1.018 (1.018, 1.019) | < 1.00 x 10^-300^ | 1.021 (1.020, 1.022) | < 1.00 x 10^-300^ |
|  |  | Age and TDI | 0.76 (0.01) | 0.18 | < 1.00 x 10^-300^ | 1.019 (1.018, 1.019) | < 1.00 x 10^-300^ | 1.022 (1.021, 1.023) | < 1.00 x 10^-300^ |
|  |  | Incl. comorbidities * | 0.76 (0.01) | 0.18 | < 1.00 x 10^-300^ | 1.022 (1.021, 1.023) | < 1.00 x 10^-300^ | 1.024 (1.023, 1.025) | < 1.00 x 10^-300^ |
|  | Spirits | Unadjusted | 0.71 (0.03) | 0.06 | 9.06 x 10^-130^ | 1.017 (1.016, 1.019) | 3.53 x 10^-78^ | 1.014 (1.011, 1.017) | 1.58 x 10^-246^ |
|  |  | Age and TDI | 0.69 (0.03) | 0.06 | 5.46 x 10^-122^ | 1.017 (1.015, 1.018) | 9.76 x 10^-71^ | 1.012 (1.009, 1.015) | 2.19 x 10^-17^ |
|  |  | Incl. comorbidities * | 0.53 (0.03) | 0.04 | 6.82 x 10^-90^ | 1.014 (1.012, 1.016) | 4.91 x 10^-46^ | 1.009 (1.005, 1.012) | 3.47 x 10^-7^ |
|  | Wine | Unadjusted | 0.23 (0.01) | 0.05 | 2.93 x 10^-103^ | 1.005 (1.004, 1.006) | 3.25 x 10^-47^ | 1.001 (0.999, 1.002) | 0.37 |
|  |  | Age and TDI | 0.24 (0.01) | 0.05 | 5.83 x 10^-107^ | 1.005 (1.005, 1.006) | 7.76 x 10^-52^ | 1.001 (0.999, 1.002) | 0.31 |
|  |  | Incl. comorbidities * | 0.29 (0.01) | 0.07 | 5.17 x 10^-204^ | 1.008 (1.007, 1.009) | 3.11 x 10^-97^ | 1.003 (1.001, 1.004) | 7.52 x 10^-4^ |
|  | | | *N* = 192,476 | | | *N* = 192,476 | | *N* = 202,703 | |
| Women | All alcohol | Unadjusted | 0.28 (0.01) | 0.06 | 1.13 x 10^-149^ | 1.007 (1.005, 1.009) | 1.01 x 10^-14^ | 1.001 (0.993, 1.007) | 0.96 |
|  |  | Age and TDI | 0.38 (0.01) | 0.08 | 2.50 x 10^-275^ | 1.011 (1.009, 1.012) | 2.02 x 10^-34^ | 1.006 (1.000, 1.013) | 0.05 |
|  |  | Incl. comorbidities * | 0.53 (0.01) | 0.11 | < 1.00 x 10^-300^ | 1.020 (1.018, 1.022) | 3.25 x 10^-112^ | 1.013 (1.007, 1.019) | 9.00 x 10^-6^ |
|  | Beer | Unadjusted | 0.67 (0.04) | 0.04 | 5.38 x 10^-74^ | 1.025 (1.021, 1.030) | 1.55 x 10^-28^ | 1.020 (1.005, 1.035) | 0.01 |
|  |  | Age and TDI | 1.03 (0.04) | 0.06 | 2.43 x 10^-175^ | 1.035 (1.030, 1.039) | 2.27 x 10^-52^ | 1.029 (1.016, 1.042) | 1.80 x 10^-5^ |
|  |  | Incl. comorbidities * | 0.96 (0.03) | 0.06 | 3.25 x 10^-213^ | 1.043 (1.038, 1.048) | 2.18 x 10^-66^ | 1.033 (1.019, 1.048) | 7.00 x 10^-6^ |
|  | Spirits | Unadjusted | 1.44 (0.04) | 0.08 | 8.94 x 10^-258^ | 1.041 (1.036, 1.045) | 4.67 x 10^-77^ | 1.032 (1.020, 1.044) | 7.39 x 10^-8^ |
|  |  | Age and TDI | 1.36 (0.04) | 0.07 | 5.59 x 10^-241^ | 1.040 (1.036, 1.045) | 5.21 x 10^-73^ | 1.031 (1.018, 1.044) | 1.00 x 10^-6^ |
|  |  | Incl. comorbidities * | 0.87 (0.04) | 0.05 | 3.84 x 10^-136^ | 1.035 (1.030, 1.040) | 2.30 x 10^-43^ | 1.024 (1.010, 1.039) | 0.001 |
|  | Wine | Unadjusted | 0.15 (0.01) | 0.03 | 1.24 x 10^-38^ | 0.999 (0.997, 1.001) | 0.30 | 0.991 (0.983, 0.999) | 0.04 |
|  |  | Age and TDI | 0.23 (0.01) | 0.05 | 1.86 x 10^-91^ | 1.003 (1.001, 1.005) | 0.01 | 0.998 (0.991, 1.006) | 0.68 |
|  |  | Incl. comorbidities * | 0.44 (0.01) | 0.09 | < 1.00 x 10^-300^ | 1.015 (1.013, 1.018) | 1.52 x 10^-50^ | 1.009 (1.002, 1.016) | 0.01 |

*Abbreviations*: β, unstandardised beta coefficient per unit/week increase in alcohol intake; SE, standard error; B, standardised beta coefficient per unit/week increase in alcohol intake; OR, odds ratio per unit/week increase in alcohol intake; 95% CI, 95% confidence interval; TDI, Townsend deprivation index; Incl., including.

*Adjusted for age, gender (all participants only), Townsend deprivation index, BMI, diuretics use, eGFR, high cholesterol, hypertension, cardiac problem, peripheral vascular disease, stroke, diabetes mellitus, and smoking status.

Bonferroni-corrected experiment-wide significance for each analysis: *P* < 0.01.

**Table S4. Details from relevant GWAS of lead SNPs at genome-wide significance included in interaction analysis.**

| **Phenotype (Study)** | **Gene (HGNC)** | *GCKR* | *KLB* | *ADH1B* | *Intergenic* | *NQO1-DT* |
| --- | --- | --- | --- | --- | --- | --- |
|  | **SNP** | rs1260326 | rs11940694 | rs1229984 | rs6460047 | rs113441031 |
|  | **Chr: Pos (b37)** | 2: 27730940 | 4: 39414993 | 4: 100239319 | 7: 73042443 | 16: 69763280 |
| Log grams per day (combined analysis)  (Schumann et al., 2016 [1]) | Effect/other alleles (effect allele freq.) | - | A/G (0.42) | - | - | - |
|  | Beta (effect allele) | - | -0.014 | - | - | - |
|  | SE | - | 0.002 | - | - | - |
|  | *P-*value | - | 9.2E-12 | - | - | - |
| Drinks per week (non-Hispanic whites)  (Jorgenson et al., 2017 [2]) | Effect allele (effect allele freq.) | - | - | T (0.05) | - | - |
|  | Beta (effect allele) | - | - | -0.19 | - | - |
|  | 95% CI | - | - | -0.22, -0.16 | - | - |
|  | *P*-value | - | - | 1.91E-35 | - | - |
| Units per week  (Clarke et al., 2017 [3]) | A1/A2 (A1 freq.) | T/C (0.39) | A/G (0.39) | - | - | - |
|  | Beta (A1) | -0.028 | -0.027 | - | - | - |
|  | SE | 0.003 | 0.003 | - | - | - |
|  | *P*-value | 1.34E-21 | 8.4E-19 | - | - | - |
| Drinks/week  (Liu et al., 2019 [4]) | REF/ALT allele (ALT freq.) | T/C (0.60) | A/G (0.60) | T/C (0.95) | T/C (0.21) | - |
|  | Beta (ALT) | 0.024 | 0.028 | 0.19 | 0.012 | - |
|  | SE | 0.002 | 0.002 | 0.006 | 0.002 | - |
|  | *P*-value | 3.33E-33 | 3.11E-46 | 1.60E-203 | 9.69E-11 | - |
| Maximum habitual alcohol intake  (Gelernter et al., 2019 [5]) | REF/ALT allele (ALT freq.) | - | - | T/C (0.97) | - | - |
|  | Beta (ALT allele) | - | - | 0.36 | - | - |
|  | SE | - | - | 0.025 | - | - |
|  | *P*-value | - | - | 4.91E-47 | - | - |
| Heavy drinker status  (Thompson et al., 2020 [6]) | A1/A0 (A1 freq.) | T/C (0.39) | A/G (0.39) | T/C (0.02) | - | - |
|  | OR (A1) | 0.94 | 0.94 | 0.63 | - | - |
|  | 95% CI | 0.92, 0.96 | 0.92, 0.96 | 0.59, 0.68 | - | - |
|  | *P*-value | 2.6E-8 | 2.1E-9 | 3.3E-36 | - | - |
| Dietary habit  (Cole et al., 2020 [7]) | Effect/ALT allele (ALT freq.) | T/C (0.61) | A/G (0.61) | T/C (0.97) | - | C/T (0.17) |
|  | Beta (effect allele) | -0.036 | -0.036 | -0.23 | - | 0.019 |
|  | SE | 0.002 | 0.002 | 0.007 | - | 0.003 |
|  | *P*-value | 2.60E-64 | 6.50E-62 | 3.80E-248 | - | 1.00E-11 |
|  | Top dietary habit | Total drinks of alcohol per month | Total drinks of alcohol per month | Total drinks of alcohol per month | - | Overall alcohol intake |

| **Phenotype (Study)** | **Gene (HGNC)** | *GCKR* | *KLB* | *ADH1B* | *Intergenic* | *NQO1-DT* |
| --- | --- | --- | --- | --- | --- | --- |
|  | **SNP** | rs1260326 | rs11940694 | rs1229984 | rs6460047 | rs113441031 |
|  | **Chr: Pos (b37)** | 2: 27730940 | 4: 39414993 | 4: 100239319 | 7: 73042443 | 16: 69763280 |
| Urate  (Tin et al., 2019 [8]) | A1/A2 (A1 freq.) | T/C (0.40) | A/G (0.46) | - | T/C (0.79) | T/C (0.16) |
|  | Effect (A1) | 0.070 | -0.017 | - | 0.039 | 0.042 |
|  | SE | 0.004 | 0.003 | - | 0.005 | 0.005 |
|  | *P*-value | 4.61E-69 | 2.37E-8 | - | 3.24E-16 | 2.90E-15 |
| Gout  (Tin et al., 2019 [8]) | A1/A2 (A1 freq.) | T/C (0.39) | - | T/C (0.03) | T/C (0.79) | T/C (0.17) |
|  | Effect (A1) | 0.19 | - | 0.38 | 0.11 | 0.11 |
|  | SE | 0.013 | - | 0.040 | 0.016 | 0.017 |
|  | *P*-value | 6.40E-45 | - | 1.27E-21 | 3.47E-12 | 1.53E-10 |

*Abbreviations*: HGNC, Hugo Gene Nomenclature Committee label; SNP, single nucleotide polymorphism; Chr, chromosome number; Pos (b37), position using build 37; freq., frequency; SE, standard error; 95% CI, 95% confidence interval; A, allele (followed by number used in corresponding GWAS for labelling the allele); REF, reference allele; ALT, alternate allele; OR, odds ratio.

References

1. Schumann G, Liu C, O’Reilly P, Gao H, Song P, Xu B, et al. KLB is associated with alcohol drinking, and its gene product β-Klotho is necessary for FGF21 regulation of alcohol preference. *Proc Natl Acad Sci U S A*. 2016;113(50):14372-7.
2. Jorgenson E, Thai KK, Hoffmann TJ, Sakoda LC, Kvale MN, Banda Y, et al. Genetic contributors to variation in alcohol consumption vary by race/ethnicity in a large multi-ethnic genome-wide association study. *Mol Psychiatry*. 2017;22(9):1359-67.
3. Clarke TK, Adams MJ, Davies G, Howard DM, Hall LS, Padmanabhan S, et al. Genome-wide association study of alcohol consumption and genetic overlap with other health-related traits in UK Biobank (N = 112 117). *Mol Psychiatry*. 2017;22(10):1376-84.
4. Liu M, Jiang Y, Wedow R, Li Y, Brazel DM, Chen F, et al. Association studies of up to 1.2 million individuals yield new insights into the genetic etiology of tobacco and alcohol use. *Nat Genet*. 2019;51(2):237-44.
5. Gelernter J, Sun N, Polimanti R, Pietrzak R, Levey DF, Lu Q, et al. Genome-wide association study of maximum habitual alcohol intake in >140,000 U.S. European and African American veterans yields novel risk loci. *Biol Psychiatry*. 2019;86(5):365-76.
6. Thompson A, Cook J, Choquet H, Jorgenson E, Yin J, Kinnunen T, et al. Functional validity, role, and implications of heavy alcohol consumption genetic loci. *Sci Adv*. 2020;6(3):eaay5034.
7. Cole JB, Florez JC, Hirschhorn JN. Comprehensive genomic analysis of dietary habits in UK Biobank identifies hundreds of genetic associations. *Nat Commun*. 2020;11(1):1467.

**Table S5. Interaction terms between urate-associated SNPs and all alcohol intake (units/week) for serum urate level (μmol/L) using other models of adjustment for covariables.**

| ***Locus***  **SNP** | **Urate-increasing allele** | **Adjustment** | **All participants (*N* = 431,555)** | | | | **Men (*N* = 195,068)** | | | | **Women (*N* = 236,487)** | | | |
| --- | --- | --- | --- | --- | --- | --- | --- | --- | --- | --- | --- | --- | --- | --- |
|  |  |  | **Obs, %** | **IT (β)** | **SE** | **P-value** | **Obs, %** | **IT (β)** | **SE** | ***P*-value** | **Obs, %** | **IT (β)** | **SE** | ***P*-value** |
| *GCKR*  rs1260326 | T | Unadjusted | 83.8% | 0.013 | 0.014 | 0.33 | 88.1% | 0.011 | 0.015 | 0.47 | 80.3% | -0.060 | 0.022 | 0.01 |
|  |  | Age, gender, TDI | 83.7% | 0.006 | 0.012 | 0.63 | 88.0% | 0.011 | 0.015 | 0.46 | 80.2% | -0.053 | 0.022 | 0.02 |
|  |  | Incl. comorbidities * | 83.1% | 0.013 | 0.010 | 0.21 | 87.3% | 0.013 | 0.013 | 0.33 | 79.6% | -0.036 | 0.018 | 0.05 |
| *KLB*  rs11940694 | G | Unadjusted | 81.2% | -0.023 | 0.019 | 0.23 | 85.4% | -0.038 | 0.021 | 0.07 | 77.8% | 0.011 | 0.031 | 0.74 |
|  |  | Age, gender, TDI | 81.1% | -0.015 | 0.016 | 0.35 | 85.3% | -0.039 | 0.021 | 0.07 | 77.7% | 0.009 | 0.031 | 0.76 |
|  |  | Incl. comorbidities * | 80.6% | -0.018 | 0.014 | 0.19 | 84.7% | -0.023 | 0.019 | 0.22 | 77.1% | -0.033 | 0.026 | 0.20 |
| *ADH1B*  rs1229984 | T | Unadjusted | 83.8% | 0.463 | 0.039 | 1.02 x 10^-32^ | 88.1% | 0.289 | 0.044 | 5.83 x 10^-11^ | 80.3% | 0.319 | 0.061 | 2.08 x 10^-7^ |
|  |  | Age, gender, TDI | 83.7% | 0.313 | 0.034 | 1.17 x 10^-20^ | 88.0% | 0.290 | 0.044 | 4.43 x 10^-11^ | 80.2% | 0.312 | 0.060 | 2.09 x 10^-7^ |
|  |  | Incl. comorbidities * | 83.1% | 0.424 | 0.029 | 2.89 x 10^-48^ | 87.3% | 0.355 | 0.039 | 7.87 x 10^-20^ | 79.6% | 0.556 | 0.050 | 3.96 x 10^-28^ |
| *MLXIPL*  rs6460047 | T | Unadjusted | 83.3% | 0.077 | 0.032 | 0.02 | 87.6% | 0.096 | 0.036 | 0.01 | 79.8% | 0.088 | 0.055 | 0.11 |
|  |  | Age, gender, TDI | 83.2% | 0.101 | 0.028 | 2.61 x 10^-4^ | 87.5% | 0.096 | 0.036 | 0.01 | 79.7% | 0.106 | 0.053 | 0.05 |
|  |  | Incl. comorbidities * | 82.7% | 0.094 | 0.024 | 8.75 x 10^-5^ | 86.9% | 0.103 | 0.031 | 9.96 x 10^-4^ | 79.2% | 0.060 | 0.045 | 0.18 |
| *NFAT5*  rs113441031 | T | Unadjusted | 83.2% | -0.010 | 0.014 | 0.47 | 87.5% | -0.010 | 0.016 | 0.52 | 79.7% | 0.034 | 0.024 | 0.15 |
|  |  | Age, gender, TDI | 83.1% | 0.009 | 0.012 | 0.46 | 87.3% | -0.009 | 0.016 | 0.59 | 79.6% | 0.033 | 0.023 | 0.15 |
|  |  | Incl. comorbidities * | 82.5% | 0.017 | 0.011 | 0.12 | 86.7% | 0.006 | 0.014 | 0.67 | 79.0% | 0.026 | 0.019 | 0.18 |

*Abbreviations*: SNP, single nucleotide polymorphism; Obs, observations (%); IT, interaction term for SNP (≥one urate-raising allele vs. none) × all alcohol intake (units/week); β, unstandardised beta coefficient; SE, standard error; TDI, Townsend deprivation index; Incl., including.

*Adjusted for age, gender (all participants only), Townsend deprivation index, BMI, diuretics use, eGFR, high cholesterol, hypertension, cardiac problem, peripheral vascular disease, stroke, diabetes mellitus, and smoking status.

Bonferroni-corrected experiment-wide significance for each analysis: *P* < 0.01.

**Table S6. Interaction terms between urate-associated SNPs and all alcohol intake (units/week) for hyperuricaemia using other models of adjustment for covariables.**

| ***Locus***  **SNP** | **Urate-increasing allele** | **Adjustment** | **All participants (*N* = 431,555)** | | | | **Men (*N* = 195,068)** | | | | **Women (*N* = 236,487)** | | | |
| --- | --- | --- | --- | --- | --- | --- | --- | --- | --- | --- | --- | --- | --- | --- |
|  |  |  | **Obs, %** | **IT (OR)** | **95% CI** | ***P*-value** | **Obs, %** | **IT (OR)** | **95% CI** | ***P*-value** | **Obs, %** | **IT (OR)** | **95% CI** | ***P*-value** |
| *GCKR*  rs1260326 | T | Unadjusted | 83.8% | 1.000 | 0.999, 1.001 | 0.74 | 88.1% | 1.000 | 0.999, 1.001 | 0.93 | 80.3% | 0.997 | 0.994, 1.001 | 0.14 |
|  |  | Age, gender, TDI | 83.7% | 1.000 | 0.999, 1.001 | 0.57 | 88.0% | 1.000 | 0.999, 1.001 | 0.97 | 80.2% | 0.998 | 0.994, 1.001 | 0.22 |
|  |  | Incl. comorbidities * | 83.1% | 1.000 | 0.999, 1.001 | 0.51 | 87.3% | 1.000 | 0.999, 1.001 | 0.88 | 79.6% | 0.998 | 0.995, 1.002 | 0.33 |
| *KLB*  rs11940694 | G | Unadjusted | 81.2% | 0.999 | 0.998, 1.001 | 0.37 | 85.4% | 0.999 | 0.998, 1.001 | 0.45 | 77.8% | 0.996 | 0.991, 1.001 | 0.13 |
|  |  | Age, gender, TDI | 81.1% | 0.999 | 0.998, 1.001 | 0.31 | 85.3% | 0.999 | 0.998, 1.001 | 0.41 | 77.7% | 0.996 | 0.992, 1.001 | 0.15 |
|  |  | Incl. comorbidities * | 80.6% | 1.000 | 0.998, 1.001 | 0.66 | 84.7% | 1.000 | 0.998, 1.001 | 0.96 | 77.1% | 0.995 | 0.990, 0.999 | 0.03 |
| *ADH1B*  rs1229984 | T | Unadjusted | 83.8% | 1.009 | 1.007, 1.012 | 3.77 x 10^-13^ | 88.1% | 1.006 | 1.004, 1.009 | 5.10 x 10^-6^ | 80.3% | 1.012 | 1.003, 1.021 | 0.01 |
|  |  | Age, gender, TDI | 83.7% | 1.007 | 1.005, 1.010 | 3.54 x 10^-8^ | 88.0% | 1.007 | 1.004, 1.009 | 3.24 x 10^-6^ | 80.2% | 1.012 | 1.003, 1.020 | 0.01 |
|  |  | Incl. comorbidities * | 83.1% | 1.011 | 1.008, 1.014 | 1.88 x 10^-14^ | 87.3% | 1.009 | 1.006, 1.012 | 5.41 x 10^-10^ | 79.6% | 1.022 | 1.014, 1.031 | 2.89 x 10^-7^ |
| *MLXIPL*  rs6460047 | T | Unadjusted | 83.3% | 1.003 | 1.000, 1.005 | 0.02 | 87.6% | 1.002 | 1.000, 1.004 | 0.07 | 79.8% | 1.015 | 1.004, 1.027 | 0.01 |
|  |  | Age, gender, TDI | 83.2% | 1.003 | 1.001, 1.005 | 0.01 | 87.5% | 1.002 | 1.000, 1.005 | 0.06 | 79.7% | 1.015 | 1.004, 1.026 | 0.01 |
|  |  | Incl. comorbidities * | 82.7% | 1.003 | 1.001, 1.006 | 0.01 | 86.9% | 1.003 | 1.000, 1.005 | 0.03 | 79.2% | 1.014 | 1.003, 1.024 | 0.01 |
| *NFAT5*  rs113441031 | T | Unadjusted | 83.2% | 1.000 | 0.999, 1.001 | 0.41 | 87.5% | 0.999 | 0.998, 1.000 | 0.18 | 79.7% | 1.004 | 1.000, 1.008 | 0.04 |
|  |  | Age, gender, TDI | 83.1% | 1.000 | 0.999, 1.001 | 0.57 | 87.3% | 0.999 | 0.998, 1.000 | 0.22 | 79.6% | 1.004 | 1.000, 1.007 | 0.05 |
|  |  | Incl. comorbidities * | 82.5% | 1.000 | 0.999, 1.001 | 0.93 | 86.7% | 1.000 | 0.999, 1.001 | 0.59 | 79.0% | 1.002 | 0.999, 1.006 | 0.25 |

*Abbreviations*: SNP, single nucleotide polymorphism; Obs, observations (%); IT, interaction term for SNP (≥one urate-raising allele vs. none) × all alcohol intake (units/week); OR, odds ratio; 95% CI, 95% confidence interval; TDI, Townsend deprivation index; Incl., including.

*Adjusted for age, gender (all participants only), Townsend deprivation index, BMI, diuretics use, eGFR, high cholesterol, hypertension, cardiac problem, peripheral vascular disease, stroke, diabetes mellitus, and smoking status.

Bonferroni-corrected experiment-wide significance for each analysis: *P* < 0.01.

**Table S7. Interaction terms between *ADH1B* or *MLXIPL* and beer, spirits, and wine intake (units/week) for serum urate level (μmol/L).** Significant interactions highlighted in bold.

| ***Locus***  **SNP** | **Urate-increasing/gout risk allele** | **Alcoholic beverage type** | **All participants** | | | | **Men** | | | | **Women** | | | |
| --- | --- | --- | --- | --- | --- | --- | --- | --- | --- | --- | --- | --- | --- | --- |
|  |  |  | ***N*** | **IT (β)** | **SE** | ***P*-value** | ***N*** | **IT (β)** | **SE** | ***P*-value** | ***N*** | **IT (β)** | **SE** | ***P*-value** |
| *ADH1B*  rs1229984 | T | Beer | 345,339 | 0.50 | 0.05 | **6.20 x 10^-24^** | 163,469 | 0.44 | 0.06 | **9.79 x 10^-14^** | 181,870 | 0.49 | 0.20 | 0.01 |
|  |  | Spirits |  | 0.65 | 0.12 | **1.37 x 10^-7^** |  | 0.40 | 0.16 | 0.01 |  | 1.04 | 0.20 | **1.62 x 10^-7^** |
|  |  | Wine |  | 0.39 | 0.04 | **1.34 x 10^-24^** |  | 0.28 | 0.05 | **2.32 x 10^-7^** |  | 0.52 | 0.05 | **1.74 x 10^-21^** |
| *MLXIPL*  rs6460047 | T | Beer | 343,465 | 0.17 | 0.04 | **6.86 x 10^-6^** | 162,589 | 0.15 | 0.04 | **4.62 x 10^-4^** | 180,876 | 0.28 | 0.15 | 0.06 |
|  |  | Spirits |  | 0.31 | 0.10 | **0.001** |  | 0.28 | 0.12 | 0.02 |  | 0.31 | 0.17 | 0.06 |
|  |  | Wine |  | 0.02 | 0.03 | 0.54 |  | 0.02 | 0.05 | 0.69 |  | 0.03 | 0.05 | 0.53 |

*Abbreviations*: SNP, single nucleotide polymorphism; IT, interaction term for SNP (≥one urate-increasing or gout risk allele vs. none) × all alcohol intake (units/week); β, unstandardised beta coefficient; SE, standard error.

Adjusted for age, gender (all participants only), Townsend deprivation index, BMI, diuretics use, eGFR, high cholesterol, hypertension, cardiac problem, peripheral vascular disease, stroke, diabetes mellitus, smoking status, other alcoholic beverage types (beer/spirits/wine/other) intake, meat intake, fish intake, coffee intake, tea intake, fruit intake, vegetable intake, bread intake, cereal intake, and cheese intake.

Bonferroni-corrected experiment-wide significance for each analysis: *P* < 0.008.

**Table S8. Interaction terms between *ADH1B* and beer, spirits, and wine intake (units/week) for hyperuricaemia and gout.** Significant interactions highlighted in bold.

| ***Locus***  **SNP** | **Urate-increasing/gout risk allele** | **Alcoholic beverage type** | **All participants** | | | | **Men** | | | | **Women** | | | |
| --- | --- | --- | --- | --- | --- | --- | --- | --- | --- | --- | --- | --- | --- | --- |
|  |  |  | ***N*** | **IT (OR)** | **95% CI** | ***P*-value** | ***N*** | **IT (OR)** | **95% CI** | ***P*-value** | ***N*** | **IT (OR)** | **95% CI** | ***P*-value** |
| ***Hyperuricaemia*** | | | | | | | | | | | | | | |
| *ADH1B*  rs1229984 | T | Beer | 345,339 | 1.013 | 1.008, 1.017 | **1.34 x 10^-8^** | 163,469 | 1.012 | 1.008, 1.017 | **1.18 x 10^-7^** | 181,870 | 1.012 | 0.976, 1.049 | 0.51 |
|  |  | Spirits |  | 1.014 | 1.003, 1.025 | **0.01** |  | 1.009 | 0.997, 1.021 | 0.14 |  | 1.038 | 1.008, 1.069 | **0.01** |
|  |  | Wine |  | 1.009 | 1.005, 1.013 | **2.89 x 10^-6^** |  | 1.007 | 1.003, 1.011 | **9.23 x 10^-4^** |  | 1.022 | 1.012, 1.032 | **1.16 x 10^-5^** |
| ***Gout*** | | | | | | | | | | | | | | |
| *ADH1B*  rs1229984 | T | Beer | 350,057 | 1.013 | 1.008, 1.019 | **3.81 x 10^-6^** | 167,653 | 1.013 | 1.007, 1.019 | **9.48 x 10^-6^** | 182,404 | 1.061 | 1.003, 1.122 | 0.04 |
|  |  | Spirits |  | 1.012 | 0.995, 1.030 | 0.16 |  | 1.014 | 0.996, 1.032 | 0.13 |  | 0.986 | 0.887, 1.096 | 0.80 |
|  |  | Wine |  | 1.013 | 1.007, 1.019 | **1.80 x 10^-5^** |  | 1.012 | 1.006, 1.018 | **1.64 x 10^-4^** |  | 1.031 | 1.007, 1.055 | **0.01** |

*Abbreviations*: SNP, single nucleotide polymorphism; IT, interaction term for SNP (≥one urate-increasing or gout risk allele vs. none) × all alcohol intake (units/week); OR, odds ratio; 95% CI, 95% confidence interval.

Adjusted for age, gender (all participants only), Townsend deprivation index, BMI, diuretics use, eGFR, high cholesterol, hypertension, cardiac problem, peripheral vascular disease, stroke, diabetes mellitus, smoking status, other alcoholic beverage types (beer/spirits/wine/other) intake, meat intake, fish intake, coffee intake, tea intake, fruit intake, vegetable intake, bread intake, cereal intake, and cheese intake.

Bonferroni-corrected experiment-wide significance for each analysis: *P* < 0.02.

**Table S9. Interaction terms between gout-associated SNPs and all alcohol intake (units/week) for gout using other models of adjustment for covariables.**

| ***Locus***  **SNP** | **Gout risk allele** | **Adjustment** | **All participants (*N* = 458,367)** | | | | **Men (*N* = 209,493)** | | | | **Women (*N* = 248,874)** | | | |
| --- | --- | --- | --- | --- | --- | --- | --- | --- | --- | --- | --- | --- | --- | --- |
|  |  |  | **Obs, %** | **IT (OR)** | **95% CI** | ***P*-value** | **Obs, %** | **IT (OR)** | **95% CI** | ***P*-value** | **Obs, %** | **IT (OR)** | **95% CI** | ***P*-value** |
| *GCKR*  rs1260326 | T | Unadjusted | 83.9% | 1.000 | 0.999, 1.002 | 0.61 | 88.2% | 1.000 | 0.998, 1.002 | 0.89 | 80.3% | 0.994 | 0.980, 1.008 | 0.37 |
|  |  | Age, gender, TDI | 83.8% | 1.000 | 0.998, 1.002 | 0.96 | 88.1% | 1.000 | 0.998, 1.002 | 0.94 | 80.2% | 0.995 | 0.982, 1.008 | 0.45 |
|  |  | Incl. comorbidities * | 79.3% | 1.000 | 0.998, 1.002 | 0.78 | 83.4% | 1.000 | 0.998, 1.002 | 0.81 | 75.9% | 0.997 | 0.985, 1.008 | 0.56 |
| *ADH1B*  rs1229984 | T | Unadjusted | 83.9% | 1.012 | 1.008, 1.015 | 6.16 x 10^-10^ | 88.2% | 1.010 | 1.006, 1.014 | 1.57 x 10^-6^ | 80.3% | 1.020 | 0.996, 1.044 | 0.10 |
|  |  | Age, gender, TDI | 83.8% | 1.010 | 1.006, 1.014 | 3.10 x 10^-7^ | 88.1% | 1.010 | 1.006, 1.014 | 6.60 x 10^-7^ | 80.2% | 1.018 | 0.995, 1.041 | 0.12 |
|  |  | Incl. comorbidities * | 79.3% | 1.013 | 1.009, 1.017 | 1.15 x 10^-10^ | 83.4% | 1.013 | 1.009, 1.017 | 1.29 x 10^-9^ | 75.9% | 1.026 | 1.006, 1.047 | 0.01 |
| *MLXIPL* rs6460047 | T | Unadjusted | 83.5% | 1.000 | 0.996, 1.004 | 0.90 | 87.7% | 1.000 | 0.996, 1.005 | 0.88 | 79.9% | 0.973 | 0.953, 0.993 | 0.02 |
|  |  | Age, gender, TDI | 83.4% | 1.000 | 0.996, 1.004 | 0.93 | 87.6% | 1.000 | 0.996, 1.004 | 0.98 | 79.8% | 0.975 | 0.955, 0.995 | 0.02 |
|  |  | Incl. comorbidities * | 78.9% | 0.999 | 0.995, 1.004 | 0.80 | 83.0% | 1.000 | 0.995, 1.004 | 0.89 | 75.5% | 0.978 | 0.960, 0.997 | 0.02 |
| *NFAT5*  rs113441031 | T | Unadjusted | 83.3% | 0.999 | 0.997, 1.000 | 0.07 | 87.6% | 0.999 | 0.997, 1.000 | 0.11 | 79.7% | 0.995 | 0.981, 1.010 | 0.54 |
|  |  | Age, gender, TDI | 83.2% | 0.999 | 0.997, 1.000 | 0.13 | 87.5% | 0.999 | 0.997, 1.001 | 0.18 | 79.7% | 0.996 | 0.982, 1.009 | 0.54 |
|  |  | Incl. comorbidities * | 78.8% | 0.999 | 0.997, 1.001 | 0.20 | 82.8% | 0.999 | 0.997, 1.001 | 0.20 | 75.3% | 0.999 | 0.987, 1.011 | 0.86 |

*Abbreviations*: SNP, single nucleotide polymorphism; Obs, observations (%); IT, interaction term for SNP (≥one gout risk allele vs. none) × all alcohol intake (units/week); OR, odds ratio; 95% CI, 95% confidence interval; TDI, Townsend deprivation index; Incl., including.

*Adjusted for age, gender (all participants only), Townsend deprivation index, BMI, diuretics use, eGFR, high cholesterol, hypertension, cardiac problem, peripheral vascular disease, stroke, diabetes mellitus, and smoking status.

Bonferroni-corrected experiment-wide significance for each analysis: *P* < 0.01.

**Table S10. Genotype-stratified association analysis of *ADH1B* or *MLXIPL* and binarized alcohol intake for serum urate level using other models of adjustment for covariables.**

| ***Locus* SNP** | **Urate-raising allele** | ***N*** | **Adjustment** | **No alcohol intake** | | **Any alcohol intake** | | | | |  |
| --- | --- | --- | --- | --- | --- | --- | --- | --- | --- | --- | --- |
|  |  |  |  | **β (SE)** | ***P*-value** | **β (SE)** | | ***P*-value** | ***P*_difference_** | |  |
| ***All participants*** | | | | | | | | | | | |
|  | | | | *N* = 29,063 | | | *N* = 402,182 | | |  | |
| *ADH1B*  rs1229984 | T- | *N* = 409,079 | Unadjusted | 1 | - | 14.38 (0.50) | | 1.98 x 10^-179^ |  | |  |
|  |  |  | Age, gender, TDI |  |  | 7.26 (0.43) | | 2.81 x 10^-64^ |  |  |  |
|  |  |  | Incl. comorbidities * |  |  | 11.78 (0.37) | | 1.37 x 10^-218^ |  |  |  |
|  | T+ | *N* = 22,166 | Unadjusted | -1.95 (1.94) | 0.31 | 20.06 (0.74) | | 3.20 x 10^-160^ | 8.05 x 10^-23^ | |  |
|  |  |  | Age, gender, TDI | 1.67 (1.64) | 0.31 | 12.02 (0.63) | | 8.51 x 10^-81^ | 1.54 x 10^-22^ | |  |
|  |  |  | Incl. comorbidities * | 1.76 (1.42) | 0.22 | 18.01 (0.55) | | 1.12 x 10^-236^ | 2.69 x 10^-49^ | |  |
|  | | | | *N* = 28,898 | | | *N* = 400,000 | | |  | |
| *MLXIPL*  rs6460047 | T- | *N* = 18,299 | Unadjusted | 1 | - | 10.67 (2.36) | | 6.18 x 10^-6^ |  | |  |
|  |  |  | Age, gender, TDI |  |  | 3.20 (2.00) | | 0.11 |  |  |  |
|  |  |  | Incl. comorbidities * |  |  | 10.34 (1.73) | | 2.39 x 10^-9^ |  |  |  |
|  | T+ | *N* = 410,599 | Unadjusted | 0.78 (2.33) | 0.74 | 15.76 (2.28) | | 5.16 x 10^-12^ | 5.74 x 10^-16^ | |  |
|  |  |  | Age, gender, TDI | 0.23 (1.97) | 0.91 | 7.81 (1.93) | | 5.36 x 10^-5^ | 3.54 x 10^-18^ | |  |
|  |  |  | Incl. comorbidities * | 3.47 (1.71) | 0.04 | 15.54 (1.68) | | 1.74 x 10^-20^ | 9.26 x 10^-30^ | |  |
| ***Men*** | | | | | | | | | | | |
|  | | | | *N* = 9,951 | | | *N* = 184,962 | | |  | |
| *ADH1B*  rs1229984 | T- | *N* = 184,785 | Unadjusted | 1 | - | 17.70 (0.75) | | 9.58 x 10^-122^ |  | |  |
|  |  |  | Age and TDI |  |  | 18.42 (0.76) | | 2.06 x 10^-130^ |  |  |  |
|  |  |  | Incl. comorbidities * |  |  | 19.15 (0.68) | | 1.62 x 10^-176^ |  |  |  |
|  | T+ | *N* = 10,128 | Unadjusted | 3.20 (3.09) | 0.30 | 24.27 (1.03) | | 1.32 x 10^-121^ | 1.43 x 10^-18^ | |  |
|  |  |  | Age and TDI | 3.43 (3.09) | 0.27 | 24.95 (1.04) | | 1.05 x 10^-127^ | 2.23 x 10^-18^ | |  |
|  |  |  | Incl. comorbidities * | 0.84 (2.76) | 0.76 | 26.51 (0.92) | | 2.97 x 10^-181^ | 7.16 x 10^-29^ | |  |
|  | | | | *N* = 9,891 | | | *N* = 183,980 | | |  | |
| *MLXIPL*  rs6460047 | T- | *N* = 8,163 | Unadjusted | 1 | - | 11.07 (3.58) | | 0.002 |  | |  |
|  |  |  | Age and TDI |  |  | 11.77 (3.58) | | 0.001 |  |  |  |
|  |  |  | Incl. comorbidities * |  |  | 13.69 (3.18) | | 1.63 x 10^-5^ |  |  |  |
|  | T+ | *N* = 185,708 | Unadjusted | -1.07 (3.57) | 0.76 | 17.02 (3.49) | | 1.11 x 10^-6^ | 5.30 x 10^-13^ | |  |
|  |  |  | Age and TDI | -0.99 (3.56) | 0.78 | 17.81 (3.49) | | 3.45 x 10^-7^ | 2.43 x 10^-13^ | |  |
|  |  |  | Incl. comorbidities * | 0.11 (3.16) | 0.97 | 19.81 (3.10) | | 1.63 x 10^-10^ | 6.93 x 10^-17^ | |  |
| ***Women*** | | | | | | | | | | | |
|  | | | | *N* = 19,112 | | | *N* = 217,220 | | |  | |
| *ADH1B*  rs1229984 | T- | *N* = 224,294 | Unadjusted | 1 | - | -2.29 (0.51) | | 8.46 x 10^-6^ |  | |  |
|  |  |  | Age and TDI |  |  | 2.291 (0.51) | | 5.98 x 10^-6^ |  |  |  |
|  |  |  | Incl. comorbidities * |  |  | 9.04 (0.43) | | 4.37 x 10^-99^ |  |  |  |
|  | T+ | *N* = 12,038 | Unadjusted | -0.67 (1.92) | 0.73 | 0.82 (0.80) | | 0.31 | 1.43 x 10^-6^ | |  |
|  |  |  | Age and TDI | -0.02 (1.87) | 0.99 | 5.54 (0.79) | | 2.18 x 10^-12^ | 2.72 x 10^-7^ | |  |
|  |  |  | Incl. comorbidities * | 1.27 (1.57) | 0.42 | 14.18 (0.66) | | 5.18 x 10^-101^ | 4.81 x 10^-22^ | |  |
|  | | | | *N* = 19,007 | | | *N* = 216,023 | | |  | |
| *MLXIPL*  rs6460047 | T- | *N* = 10,136 | Unadjusted | 1 | - | -4.29 (2.40) | | 0.07 |  | |  |
|  |  |  | Age and TDI |  |  | -0.03 (2.35) | | 0.99 |  |  |  |
|  |  |  | Incl. comorbidities * |  |  | 9.71 (1.97) | | 8.66 x 10^-7^ |  |  |  |
|  | T+ | *N* = 224,891 | Unadjusted | 1.07 (2.35) | 0.65 | -0.89 (2.31) | | 0.70 | 9.06 x 10^-7^ | |  |
|  |  |  | Age and TDI | 0.67 (2.30) | 0.77 | 3.28 (2.25) | | 0.15 | 1.00 x 10^-6^ | |  |
|  |  |  | Incl. comorbidities * | 4.85 (1.93) | 0.01 | 14.08 (1.90) | | 1.13 x 10^-13^ | 1.92 x 10^-14^ | |  |

*Abbreviations*: SNP, single nucleotide polymorphism; OR, odds ratio of hyperuricaemia compared to reference group (no urate-raising allele × no alcohol intake); 95% CI, 95% confidence interval; *P*_difference,_ *P*-value for odds ratio of hyperuricaemia for presence vs. absence of urate-raising allele in alcohol-exposed (any alcohol intake) subgroup; TDI, Townsend deprivation index; Incl., including.

*N* is number of participants in unadjusted analyses.

*Adjusted for age, gender (all participants only), Townsend deprivation index, BMI, diuretics use, eGFR, high cholesterol, hypertension, cardiac problem, peripheral vascular disease, stroke, diabetes mellitus, and smoking status.

Bonferroni-corrected experiment-wide significance for each analysis: *P* < 0.03.

**Table S11. Genotype-stratified association analysis of *ADH1B* or *MLXIPL* and binarized beer, spirits, and wine intake for serum urate level (μmol/L).**

| ***Locus***  **SNP** | **Alcoholic beverage type** | **Urate- raising allele** | ***N*** | **No intake of alcoholic beverage type** | | **Any intake of alcoholic beverage type** | | |  |
| --- | --- | --- | --- | --- | --- | --- | --- | --- | --- |
|  |  |  |  | **β (SE)** | ***P*-value** | **β (SE)** | ***P*-value** | ***P*_difference_** |  |
| *ADH1B*  rs1229984 |  |  |  | *N* = 183,687 | | *N* = 161,652 | |  |  |
|  | Beer | T- | *N* = 328,530 | 1 | - | 9.53 (0.24) | < 1.00 x 10^-300^ |  |  |
|  |  | T+ | *N* = 16,809 | 6.79 (0.61) | 1.53 x 10^-28^ | 18.69 (0.69) | 3.86 x 10^-159^ | 1.30 x 10^-34^ |  |
|  |  |  |  | *N* = 230,266 | | *N* = 115,073 | |  |  |
|  | Spirits | T- | *N* = 328,530 | 1 | - | 3.00 (0.21) | 1.16 x 10^-44^ |  |  |
|  |  | T+ | *N* = 16,809 | 8.38 (0.55) | 1.00 x 10^-51^ | 13.33 (0.77) | 3.07 x 10^-66^ | 7.61 x 10^-37^ |  |
|  |  |  |  | *N* = 80,672 | | *N* = 264,667 | |  |  |
|  | Wine | T- | *N* = 328,530 | 1 | - | 7.13 (0.25) | 3.81 x 10^-181^ |  |  |
|  |  | T+ | *N* = 16,809 | 5.83 (0.88) | 4.61 x 10^-11^ | 16.45 (0.56) | 2.45 x 10^-188^ | 8.06 x 10^-74^ |  |
| *MLXIPL*  rs6460047 |  |  |  | *N* = 182,727 | | *N* = 160,738 | |  |  |
|  | Beer | T- | *N* = 14,719 | 1 | - | 7.31 (0.96) | 3.22 x 10^-14^ |  |  |
|  |  | T+ | *N* = 328,746 | 3.56 (0.66) | 6.23 x 10^-8^ | 13.26 (0.67) | 2.26 x 10^-86^ | 6.16 x 10^-15^ |  |
|  |  |  |  | *N* = 229,039 | | *N* = 114,426 | |  |  |
|  | Spirits | T- | *N* = 14,719 | 1 | - | 2.30 (1.00) | 0.02 |  |  |
|  |  | T+ | *N* = 328,746 | 4.61 (0.59) | 5.35 x 10^-15^ | 7.73 (0.60) | 1.20 x 10^-37^ | 3.71 x 10^-10^ |  |
|  |  |  |  | *N* = 80,222 | | *N* = 263,243 | |  |  |
|  | Wine | T- | *N* = 14,719 | 1 | - | 7.89 (1.13) | 2.67 x 10^-12^ |  |  |
|  |  | T+ | *N* = 328,746 | 5.32 (1.017) | 1.37 x 10^-7^ | 12.52 (1.00) | 4.12 x 10^-36^ | 5.91 x 10^-18^ |  |
| ***Men*** | | | | | | | | | |
| *ADH1B*  rs1229984 |  |  |  | *N* = 40,506 | | *N* = 122,963 | |  |  |
|  | Beer | T- | *N* = 155,321 | 1 | - | 13.92 (0.37) | < 1.00 x 10^-300^ |  |  |
|  |  | T+ | *N* = 8,148 | 7.87 (1.30) | 1.42 x 10^-9^ | 23.44 (0.88) | 1.60 x 10^-157^ | 5.78 x 10^-30^ |  |
|  |  |  |  | *N* = 103,501 | | *N* = 59,968 | |  |  |
|  | Spirits | T- | *N* = 155,321 | 1 | - | 3.32 (0.33) | 1.85 x 10^-24^ |  |  |
|  |  | T+ | *N* = 8,148 | 10.78 (0.87) | 6.68 x 10^-35^ | 14.92 (1.12) | 3.85 x 10^-40^ | 1.49 x 10^-23^ |  |
|  |  |  |  | *N* = 44,548 | | *N* = 118,921 | |  |  |
|  | Wine | T- | *N* = 155,321 | 1 | - | 7.78 (0.37) | 8.19 x 10^-100^ |  |  |
|  |  | T+ | *N* = 8,148 | 8.74 (1.31) | 2.93 x 10^-11^ | 18.97 (0.86) | 1.56 x 10^-107^ | 3.63 x 10^-45^ |  |
| *MLXIPL*  rs6460047 |  |  |  | *N* = 40,302 | | *N* = 122,287 | |  |  |
|  | Beer | T- | *N* = 6,887 | 1 | - | 10.67 (1.70) | 3.15 x 10^-10^ |  |  |
|  |  | T+ | *N* = 155,702 | 2.83 (1.49) | 0.06 | 16.86 (1.47) | 2.21 x 10^-30^ | 4.85 x 10^-12^ |  |
|  |  |  |  | *N* = 102,955 | | *N* = 59,634 | |  |  |
|  | Spirits | T- | *N* = 6,887 | 1 | - | 1.45 (1.52) | 0.34 |  |  |
|  |  | T+ | *N* = 155,702 | 4.97 (0.94) | 1.48 x 10^-7^ | 8.45 (0.96) | 1.46 x 10^-18^ | 1.98 x 10^-8^ |  |
|  |  |  |  | *N* = 44,316 | | *N* = 118,273 | |  |  |
|  | Wine | T- | *N* = 6,887 | 1 | - | 7.44 (1.68) | 9.01 x 10^-6^ |  |  |
|  |  | T+ | *N* = 155,702 | 5.32 (1.47) | 2.82 x 10^-4^ | 13.19 (1.45) | 1.16 x 10^-19^ | 1.79 x 10^-11^ |  |
| ***Women*** | | | | | | | | | |
| *ADH1B*  rs1229984 |  |  |  | *N* = 143,181 | | *N* = 38,689 | |  |  |
|  | Beer | T- | *N* = 173,209 | 1 | - | 5.68 (0.32) | 1.90 x 10^-72^ |  |  |
|  |  | T+ | *N* = 8,661 | 7.04 (0.65) | 2.56 x 10^-27^ | 12.27 (1.29) | 1.51 x 10^-21^ | 8.58 x 10^-7^ |  |
|  |  |  |  | *N* = 126,765 | | *N* = 55,105 | |  |  |
|  | Spirits | T- | *N* = 173,209 | 1 | - | 3.64 (0.28) | 3.70 x 10^-39^ |  |  |
|  |  | T+ | *N* = 8,661 | 6.20 (0.69) | 3.89 x 10^-19^ | 12.63 (1.05) | 5.00 x 10^-33^ | 4.16 x 10^-16^ |  |
|  |  |  |  | *N* = 36,124 | | *N* = 145,746 | |  |  |
|  | Wine | T- | *N* = 173,209 | 1 | - | 7.36 (0.33) | 1.09 x 10^-108^ |  |  |
|  |  | T+ | *N* = 8,661 | 2.61 (1.17) | 0.03 | 14.89 (0.72) | 4.45 x 10^-94^ | 3.95 x 10^-30^ |  |
| *MLXIPL*  rs6460047 |  |  |  | *N* = 142,425 | | *N* = 38,451 | |  |  |
|  | Beer | T- | *N* = 7,832 | 1 | - | 4.09 (1.48) | 0.01 |  |  |
|  |  | T+ | *N* = 173,044 | 3.67 (0.68) | 7.70 x 10^-8^ | 9.41 (0.73) | 1.89 x 10^-38^ | 1.11 x 10^-4^ |  |
|  |  |  |  | *N* = 126,084 | | *N* = 54,792 | |  |  |
|  | Spirits | T- | *N* = 7,832 | 1 | - | 4.40 (1.30) | 7.37 x 10^-4^ |  |  |
|  |  | T+ | *N* = 173,044 | 4.26 (0.73) | 4.69 x 10^-9^ | 7.95 (0.75) | 2.23 x 10^-26^ | 0.003 |  |
|  |  |  |  | *N* = 35,906 | | *N* = 144,970 | |  |  |
|  | Wine | T- | *N* = 7,832 | 1 | - | 8.59 (1.50) | 1.07 x 10^-8^ |  |  |
|  |  | T+ | *N* = 173,044 | 4.84 (1.37) | 4.15 x 10^-4^ | 12.35 (1.35) | 6.82 x 10^-20^ | 2.36 x 10^-8^ |  |

*Abbreviations*: SNP, single nucleotide polymorphism; OR, odds ratio of hyperuricaemia compared to reference group (no urate-raising allele × no alcohol intake); 95% CI, 95% confidence interval; *P*_difference_, *P*-value for odds ratio of hyperuricaemia for presence vs. absence of urate-raising allele in alcohol-exposed (any beer/spirits/wine intake) subgroup.

Adjusted for age, gender (all participants only), Townsend deprivation index, BMI, diuretics use, eGFR, high cholesterol, hypertension, cardiac problem, peripheral vascular disease, stroke, diabetes mellitus, smoking status, other alcoholic beverage types (beer/spirits/wine/other) intake, meat intake, fish intake, coffee intake, tea intake, fruit intake, vegetable intake, bread intake, cereal intake, and cheese intake.

Bonferroni-corrected experiment-wide significance for each analysis: *P* < 0.008.

**Table S12. Genotype-stratified association analysis of *ADH1B* and binarized alcohol intake for hyperuricaemia using other models of adjustment for covariables.**

| ***Locus* SNP** | **Urate-raising allele** | ***N*** | **Adjustment** | **No alcohol intake** | | **Any alcohol intake** | | | |  |
| --- | --- | --- | --- | --- | --- | --- | --- | --- | --- | --- |
|  |  |  |  | **OR (95% CI)** | ***P*-value** | **OR (95% CI)** | ***P*-value** | ***P*_difference_** | |  |
| ***All participants*** | | | | | | | | | | |
|  | | | | *N* = 29,063 | | *N* = 402,182 | | |  | |
| *ADH1B*  rs1229984 | T- | *N* = 409,079 | Unadjusted | 1 | - | 1.44 (1.38, 1.51) | 3.25 x 10^-57^ |  | |  |
|  |  |  | Age, gender, TDI |  |  | 1.24 (1.18, 1.29) | 1.12 x 10^-18^ |  |  |  |
|  |  |  | Incl. comorbidities * |  |  | 1.54 (1.46, 1.62) | 6.86 x 10^-59^ |  |  |  |
|  | T+ | *N* = 22,166 | Unadjusted | 0.98 (0.82, 1.17) | 0.80 | 1.68 (1.58, 1.79) | 1.19 x 10^-64^ | 7.04 x 10^-13^ | |  |
|  |  |  | Age, gender, TDI | 1.08 (0.90, 1.30) | 0.40 | 1.43 (1.35, 1.53) | 3.95 x 10^-29^ | 6.38 x 10^-11^ | |  |
|  |  |  | Incl. comorbidities * | 1.09 (0.89, 1.33) | 0.42 | 1.92 (1.79, 2.06) | 1.18 x 10^-76^ | 7.90 x 10^-20^ | |  |
| ***Men*** | | | | | | | | | | |
|  | | | | *N* = 9,951 | | *N* = 184,962 | | |  | |
| *ADH1B*  rs1229984 | T- | *N* = 184,785 | Unadjusted | 1 | - | 1.46 (1.38, 1.55) | 4.66 x 10^-38^ |  | |  |
|  |  |  | Age and TDI |  |  | 1.51 (1.43, 1.60) | 7.48 x 10^-45^ |  |  |  |
|  |  |  | Incl. comorbidities * |  |  | 1.76 (1.65, 1.88) | 7.68 x 10^-68^ |  |  |  |
|  | T+ | *N* = 10,128 | Unadjusted | 1.10 (0.88, 1.38) | 0.41 | 1.72 (1.60, 1.85) | 1.43 x 10^-47^ | 2.89 x 10^-11^ | |  |
|  |  |  | Age and TDI | 1.11 (0.88, 1.40) | 0.36 | 1.78 (1.66, 1.92) | 2.05 x 10^-53^ | 3.19 x 10^-11^ | |  |
|  |  |  | Incl. comorbidities * | 1.08 (0.83, 1.39) | 0.57 | 2.21 (2.04, 2.39) | 9.40 x 10^-83^ | 1.62 x 10^-17^ | |  |
| ***Women*** | | | | | | | | | | |
|  | | | | *N* = 19,112 | | *N* = 217,220 | | |  | |
| *ADH1B*  rs1229984 | T- | *N* = 224,294 | Unadjusted | 1 | - | 0.70 (0.64, 0.75) | 4.25 x 10^-20^ |  | |  |
|  |  |  | Age and TDI |  |  | 0.84 (0.77, 0.90) | 6.32 x 10^-6^ |  |  |  |
|  |  |  | Incl. comorbidities * |  |  | 1.30 (1.19, 1.42) | 1.30 x 10^-8^ |  |  |  |
|  | T+ | *N* = 12,038 | Unadjusted | 0.95 (0.71, 1.27) | 0.72 | 0.74 (0.65, 0.84) | 6.50 x 10^-6^ | 0.27 | |  |
|  |  |  | Age and TDI | 0.97 (0.73, 1.30) | 0.85 | 0.89 (0.78, 1.02) | 0.097 | 0.23 | |  |
|  |  |  | Incl. comorbidities * | 1.05 (0.75, 1.46) | 0.78 | 1.56 (1.35, 1.81) | 2.46 x 10^-9^ | 0.003 | |  |

*Abbreviations*: SNP, single nucleotide polymorphism; OR, odds ratio of hyperuricaemia compared to reference group (no urate-raising allele × no alcohol intake); 95% CI, 95% confidence interval; *P*_difference,_ *P*-value for odds ratio of hyperuricaemia for presence vs. absence of urate-raising allele in alcohol-exposed (any alcohol intake) subgroup; TDI, Townsend deprivation index; Incl., including.

*N* is number of participants in unadjusted analyses.

*Adjusted for age, gender (all participants only), Townsend deprivation index, BMI, diuretics use, eGFR, high cholesterol, hypertension, cardiac problem, peripheral vascular disease, stroke, diabetes mellitus, and smoking status.

**Table S13. Genotype-stratified association analysis of *ADH1B* and binarized beer, spirits, and wine intake for hyperuricaemia.**

| ***Locus***  **SNP** | **Alcoholic beverage type** | **Urate- raising allele** | ***N*** | **No intake of alcoholic beverage type** | | **Any intake of alcoholic beverage type** | | |  |
| --- | --- | --- | --- | --- | --- | --- | --- | --- | --- |
|  |  |  |  | **OR (95% CI)** | ***P*-value** | **OR (95% CI)** | ***P*-value** | ***P*_difference_** |  |
| ***All participants*** | | | | | | | | | |
| *ADH1B* rs1229984 |  |  |  | *N* = 183,687 | | *N* = 161,652 | |  |  |
|  | Beer | T- | *N* = 328,530 | 1 | - | 1.52 (1.48, 1.57) | 5.52 x 10^-164^ |  |  |
|  |  | T+ | *N* = 16,809 | 1.28 (1.17, 1.41) | 1.09 x 10^-7^ | 2.06 (1.93, 2.21) | 2.59 x 10^-97^ | 2.43 x 10^-19^ |  |
|  |  |  |  | *N* = 230,266 | | *N* = 115,073 | |  |  |
|  | Spirits | T- | *N* = 328,530 | 1 | - | 1.10 (1.07, 1.13) | 9.78 x 10^-13^ |  |  |
|  |  | T+ | *N* = 16,809 | 1.40 (1.31, 1.49) | 1.15 x 10^-22^ | 1.59 (1.46, 1.73) | 2.04 x 10^-27^ | 2.44 x 10^-17^ |  |
|  |  |  |  | *N* = 80,672 | | *N* = 264,667 | |  |  |
|  | Wine | T- | *N* = 328,530 | 1 | - | 1.19 (1.16, 1.23) | 4.77 x 10^-31^ |  |  |
|  |  | T+ | *N* = 16,809 | 1.35 (1.22, 1.49) | 4.23 x 10^-9^ | 1.67 (1.57, 1.79) | 3.41 x 10^-53^ | 1.94 x 10^-28^ |  |
| ***Men*** | | | | | | | | | |
| *ADH1B* rs1229984 |  |  |  | *N* = 40,506 | | *N* = 122,963 | |  |  |
|  | Beer | T- | *N* = 155,321 | 1 | - | 1.59 (1.53, 1.64) | 5.93 x 10^-158^ |  |  |
|  |  | T+ | *N* = 8,148 | 1.30 (1.16, 1.45) | 9.44 x 10^-6^ | 2.12 (1.98, 2.28) | 1.08 x 10^-97^ | 1.80 x 10^-18^ |  |
|  |  |  |  | *N* = 103,501 | | *N* = 59,968 | |  |  |
|  | Spirits | T- | *N* = 155,321 | 1 | - | 1.09 (1.06, 1.12) | 1.39 x 10^-9^ |  |  |
|  |  | T+ | *N* = 8,148 | 1.41 (1.31, 1.52) | 2.52 x 10^-20^ | 1.58 (1.45, 1.73) | 1.64 x 10^-23^ | 1.78 x 10^-15^ |  |
|  |  |  |  | *N* = 44,548 | | *N* = 118,921 | |  |  |
|  | Wine | T- | *N* = 155,321 | 1 | - | 1.21 (1.18, 1.25) | 8.57 x 10^-33^ |  |  |
|  |  | T+ | *N* = 8,148 | 1.38 (1.24, 1.54) | 4.47 x 10^-9^ | 1.71 (1.59, 1.83) | 3.20 x 10^-49^ | 1.72 x 10^-24^ |  |
| ***Women*** | | | | | | | | | |
| *ADH1B* rs1229984 |  |  |  | *N* = 143,181 | | *N* = 38,689 | |  |  |
|  | Beer | T- | *N* = 173,209 | 1 | - | 1.35 (1.25, 1.46) | 6.05 x 10^-14^ |  |  |
|  |  | T+ | *N* = 8,661 | 1.34 (1.15, 1.56) | 2.23 x 10^-4^ | 1.83 (1.36, 2.48) | 8.09 x 10^-5^ | 0.05 |  |
|  |  |  |  | *N* = 126,765 | | *N* = 55,105 | |  |  |
|  | Spirits | T- | *N* = 173,209 | 1 | - | 1.22 (1.15, 1.31) | 2.14 x 10^-9^ |  |  |
|  |  | T+ | *N* = 8,661 | 1.30 (1.10, 1.54) | 0.003 | 1.80 (1.42, 2.27) | 7.34 x 10^-7^ | 0.002 |  |
|  |  |  |  | *N* = 36,124 | | *N* = 145,746 | |  |  |
|  | Wine | T- | *N* = 173,209 | 1 | - | 1.17 (1.09, 1.27) | 3.43 x 10^-5^ |  |  |
|  |  | T+ | *N* = 8,661 | 1.16 (0.90, 1.49) | 0.26 | 1.63 (1.37, 1.94) | 3.10 x 10^-8^ | 3.50 x 10^-5^ |  |

*Abbreviations*: SNP, single nucleotide polymorphism; OR, odds ratio of hyperuricaemia compared to reference group (no urate-raising allele × no alcohol intake); 95% CI, 95% confidence interval; *P*_difference_, *P*-value for odds ratio of hyperuricaemia for presence vs. absence of urate-raising allele in alcohol-exposed (any beer/spirits/wine intake) subgroup.

Adjusted for age, gender (all participants only), Townsend deprivation index, BMI, diuretics use, eGFR, high cholesterol, hypertension, cardiac problem, peripheral vascular disease, stroke, diabetes mellitus, smoking status, other alcoholic beverage types (beer/spirits/wine/other) intake, meat intake, fish intake, coffee intake, tea intake, fruit intake, vegetable intake, bread intake, cereal intake, and cheese intake.

Bonferroni-corrected experiment-wide significance for each analysis: *P* < 0.02.

**Table S14. Genotype-stratified association analysis of *ADH1B* and binarized alcohol intake for gout using other models of adjustment for covariables.**

| ***Locus* SNP** | **Urate-raising allele** | ***N*** | **Adjustment** | **No alcohol intake** | | **Any alcohol intake** | | |
| --- | --- | --- | --- | --- | --- | --- | --- | --- |
|  |  |  |  | **OR (95% CI)** | ***P*-value** | **OR (95% CI)** | ***P*-value** | ***P*_difference_** |
| ***All participants*** | | | | | | | | |
|  | | | | *N* = 30,820 | | *N* = 427,220 | |  |
| *ADH1B*  rs1229984 | T- | *N* = 434,302 | Unadjusted | 1 | - | 1.48 (1.32, 1.66) | 2.65 x 10^-11^ |  |
|  |  |  | Age, gender, TDI |  |  | 1.28 (1.14, 1.43) | 4.30 x 10^-5^ |  |
|  |  |  | Incl. comorbidities * |  |  | 1.50 (1.33, 1.70) | 1.27 x 10^-10^ |  |
|  | T+ | *N* = 23,738 | Unadjusted | 0.93 (0.58, 1.47) | 0.74 | 2.48 (2.16, 2.85) | 6.68 x 10^-37^ | 4.32 x 10^-31^ |
|  |  |  | Age, gender, TDI | 1.03 (0.64, 1.65) | 0.90 | 2.10 (1.82, 2.42) | 1.83 x 10^-24^ | 4.05 x 10^-28^ |
|  |  |  | Incl. comorbidities * | 1.00 (0.61, 1.64) | 0.99 | 2.70 (2.33, 3.14) | 1.68 x 10^-38^ | 6.69 x 10^-36^ |
| ***Men*** | | | | | | | | |
|  | | | | *N* = 10,632 | | *N* = 198,699 | |  |
| *ADH1B*  rs1229984 | T- | *N* = 198,699 | Unadjusted | 1 | - | 1.41 (1.24, 1.62) | 4.88 x 10^-7^ |  |
|  |  |  | Age and TDI |  |  | 1.53 (1.34, 1.76) | 6.27 x 10^-10^ |  |
|  |  |  | Incl. comorbidities * |  |  | 1.77 (1.54, 2.05) | 5.91 x 10^-15^ |  |
|  | T+ | *N* = 11,073 | Unadjusted | 1.03 (0.60, 1.79) | 0.90 | 2.32 (1.98, 2.72) | 2.84 x 10^-25^ | 2.37 x 10^-26^ |
|  |  |  | Age and TDI | 1.06 (0.61, 1.83) | 0.84 | 2.52 (2.15, 2.96) | 7.38 x 10^-30^ | 2.93 x 10^-26^ |
|  |  |  | Incl. comorbidities * | 0.99 (0.56, 1.77) | 0.97 | 3.19 (2.70, 3.78) | 1.92 x 10^-41^ | 1.65 x 10^-33^ |
| ***Women*** | | | | | | | | |
|  | | | | *N* = 20,188 | | *N* = 228,521 | |  |
| *ADH1B*  rs1229984 | T- | *N* = 236,044 | Unadjusted | 1 | - | 0.46 (0.36, 0.58) | 1.02 x 10^-10^ |  |
|  |  |  | Age and TDI |  |  | 0.61 (0.48, 0.77) | 4.28 x 10^-5^ |  |
|  |  |  | Incl. comorbidities * |  |  | 0.87 (0.67, 1.11) | 0.26 |  |
|  | T+ | *N* = 12,665 | Unadjusted | 0.86 (0.35, 2.13) | 0.75 | 0.75 (0.51, 1.11) | 0.15 | 0.004 |
|  |  |  | Age and TDI | 0.89 (0.36, 2.20) | 0.80 | 1.01 (0.67, 1.47) | 0.99 | 0.004 |
|  |  |  | Incl. comorbidities * | 1.01 (0.41, 2.53) | 0.98 | 1.53 (1.01, 2.30) | 0.04 | 0.001 |

*Abbreviations*: SNP, single nucleotide polymorphism; OR, odds ratio of gout compared to reference group (no gout risk allele × no alcohol intake); 95% CI, 95% confidence interval; *P*_difference_, *P*-value for odds ratio of gout for presence vs. absence of gout risk allele in alcohol-exposed (any alcohol intake) subgroup; TDI, Townsend deprivation index; Incl., including.

*N* is number of participants in unadjusted analyses.

*Adjusted for age, gender (all participants only), Townsend deprivation index, BMI, diuretics use, eGFR, high cholesterol, hypertension, cardiac problem, peripheral vascular disease, stroke, diabetes mellitus, and smoking status.

**Table S15. Genotype-stratified association analysis of *ADH1B* and binarized beer, spirits, and wine intake for gout.**

| ***Locus***  **SNP** | **Alcoholic beverage type** | **Urate- raising allele** | ***N*** | **No intake of alcoholic beverage type** | | **Any intake of alcoholic beverage type** | | |  |
| --- | --- | --- | --- | --- | --- | --- | --- | --- | --- |
|  |  |  |  | **OR (95% CI)** | ***P*-value** | **OR (95% CI)** | ***P*-value** | ***P*_difference_** |  |
| ***All participants*** | | | | | | | | | |
| *ADH1B* rs1229984 |  |  |  | *N* = 184,877 | | *N* = 165,180 | |  |  |
|  | Beer | T- | *N* = 332,858 | 1 | - | 1.72 (1.60, 1.85) | 3.84 x 10^-49^ |  |  |
|  |  | T+ | *N* = 17,199 | 1.77 (1.45, 2.16) | 2.52 x 10^-8^ | 3.43 (3.03, 3.90) | 6.37 x 10^-82^ | 1.31 x 10^-33^ |  |
|  |  |  |  | *N* = 233,096 | | *N* = 116,961 | |  |  |
|  | Spirits | T- | *N* = 332,858 | 1 | - | 1.07 (1.01, 1.13) | 0.02 |  |  |
|  |  | T+ | *N* = 17,199 | 2.08 (1.83, 2.36) | 1.79 x 10^-29^ | 2.42 (2.08, 2.81) | 7.56 x 10^-30^ | 1.04 x 10^-24^ |  |
|  |  |  |  | *N* = 82,301 | | *N* = 267,756 | |  |  |
|  | Wine | T- | *N* = 332,858 | 1 | - | 0.98 (0.92, 1.04) | 0.45 |  |  |
|  |  | T+ | *N* = 17,199 | 1.52 (1.25, 1.85) | 1.97 x 10^-5^ | 2.37 (2.10, 2.68) | 1.37 x 10^-43^ | 4.47 x 10^-54^ |  |
| ***Men*** | | | | | | | | | |
| *ADH1B*  rs1229984 |  |  |  | *N* = 41,261 | | *N* = 126,392 | |  |  |
|  | Beer | T- | *N* = 159,148 | 1 | - | 1.79 (1.66, 1.94) | 1.05 x 10^-49^ |  |  |
|  |  | T+ | *N* = 8,505 | 1.78 (1.42, 2.24) | 5.77 x 10^-7^ | 3.53 (3.10, 4.03) | 8.28 x 10^-81^ | 3.62 x 10^-32^ |  |
|  |  |  |  | *N* = 105,982 | | *N* = 61,671 | |  |  |
|  | Spirits | T- | *N* = 159,148 | 1 | - | 1.07 (1.00, 1.13) | 0.04 |  |  |
|  |  | T+ | *N* = 8,505 | 2.08 (1.82, 2.38) | 2.49 x 10^-27^ | 2.41 (2.06, 2.82) | 3.76 x 10^-28^ | 1.55 x 10^-23^ |  |
|  |  |  |  | *N* = 45,997 | | *N* = 121,656 | |  |  |
|  | Wine | T- | *N* = 159,148 | 1 | - | 1.00 (0.94, 1.07) | 0.97 |  |  |
|  |  | T+ | *N* = 8,505 | 1.51 (1.23, 1.85) | 7.25 x 10^-5^ | 2.44 (2.15, 2.77) | 1.39 x 10^-43^ | 3.41 x 10^-51^ |  |
| ***Women*** | | | | | | | | | |
| *ADH1B*  rs1229984 |  |  |  | *N* = 143,616 | | *N* = 38,788 | |  |  |
|  | Beer | T- | *N* = 173,710 | 1 | - | 1.10 (0.83, 1.46) | 0.49 |  |  |
|  |  | T+ | *N* = 8,694 | 1.80 (1.12, 2.74) | 0.01 | 3.20 (1.55, 6.58) | 0.002 | 0.01 |  |
|  |  |  |  | *N* = 127,114 | | *N* = 55,290 | |  |  |
|  | Spirits | T- | *N* = 173,710 | 1 | - | 1.21 (0.97, 1.50) | 0.10 |  |  |
|  |  | T+ | *N* = 8,694 | 1.95 (1.25, 3.05) | 0.003 | 2.59 (1.36, 4.93) | 0.004 | 0.02 |  |
|  |  |  |  | *N* = 36,304 | | *N* = 146,100 | |  |  |
|  | Wine | T- | *N* = 173,710 | 1 | - | 0.75 (0.59, 0.94) | 0.02 |  |  |
|  |  | T+ | *N* = 8,694 | 1.56 (0.85, 2.85) | 0.15 | 1.67 (1.03, 2.71) | 0.04 | 3.78 x 10^-4^ |  |

*Abbreviations*: SNP, single nucleotide polymorphism; OR, odds ratio of gout compared to reference group (no gout risk allele × no alcohol intake); 95% CI, 95% confidence interval; *P*_difference_, *P*-value for odds ratio of gout for presence vs. absence of gout risk allele in alcohol-exposed (any beer/spirits/wine intake) subgroup.

Adjusted for age, gender (all participants only), Townsend deprivation index, BMI, diuretics use, eGFR, high cholesterol, hypertension, cardiac problem, peripheral vascular disease, stroke, diabetes mellitus, smoking status, other alcoholic beverage types (beer/spirits/wine/other) intake, meat intake, fish intake, coffee intake, tea intake, fruit intake, vegetable intake, bread intake, cereal intake, and cheese intake.

Bonferroni-corrected experiment-wide significance for each analysis: *P* < 0.02

**Figure S1. Distribution of all alcohol intake (units/week) data among all participants.**


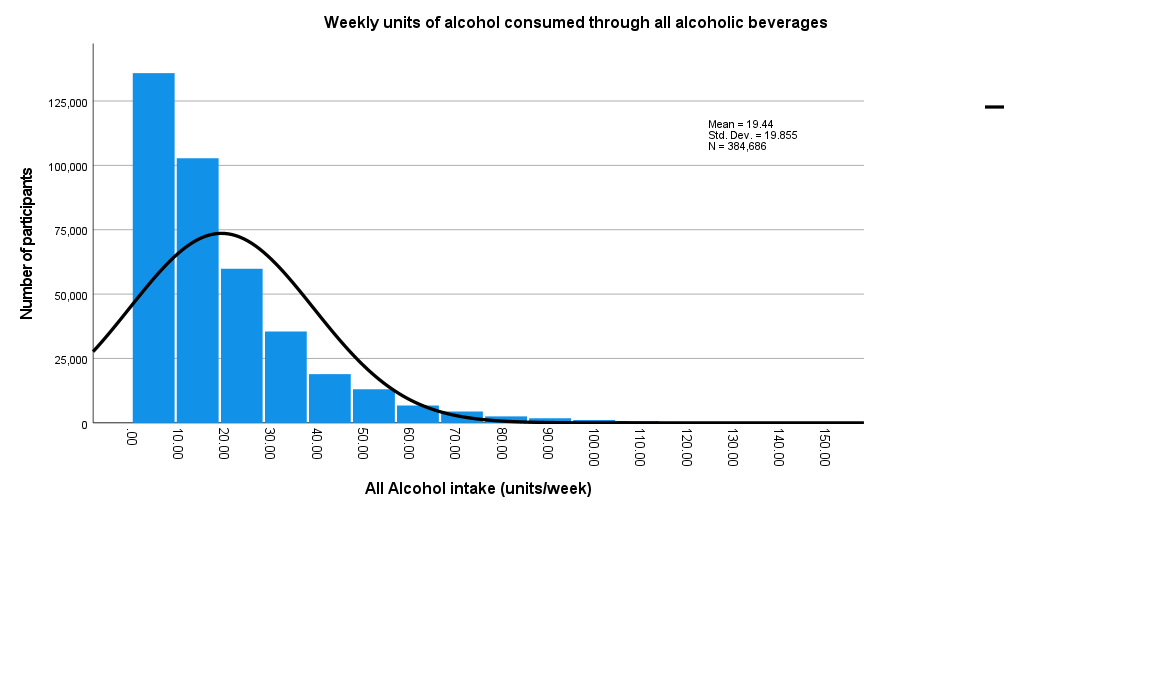


**Figure S2. LocusZoom plots within UK Biobank for five serum urate loci included in interaction analysis.**

1.
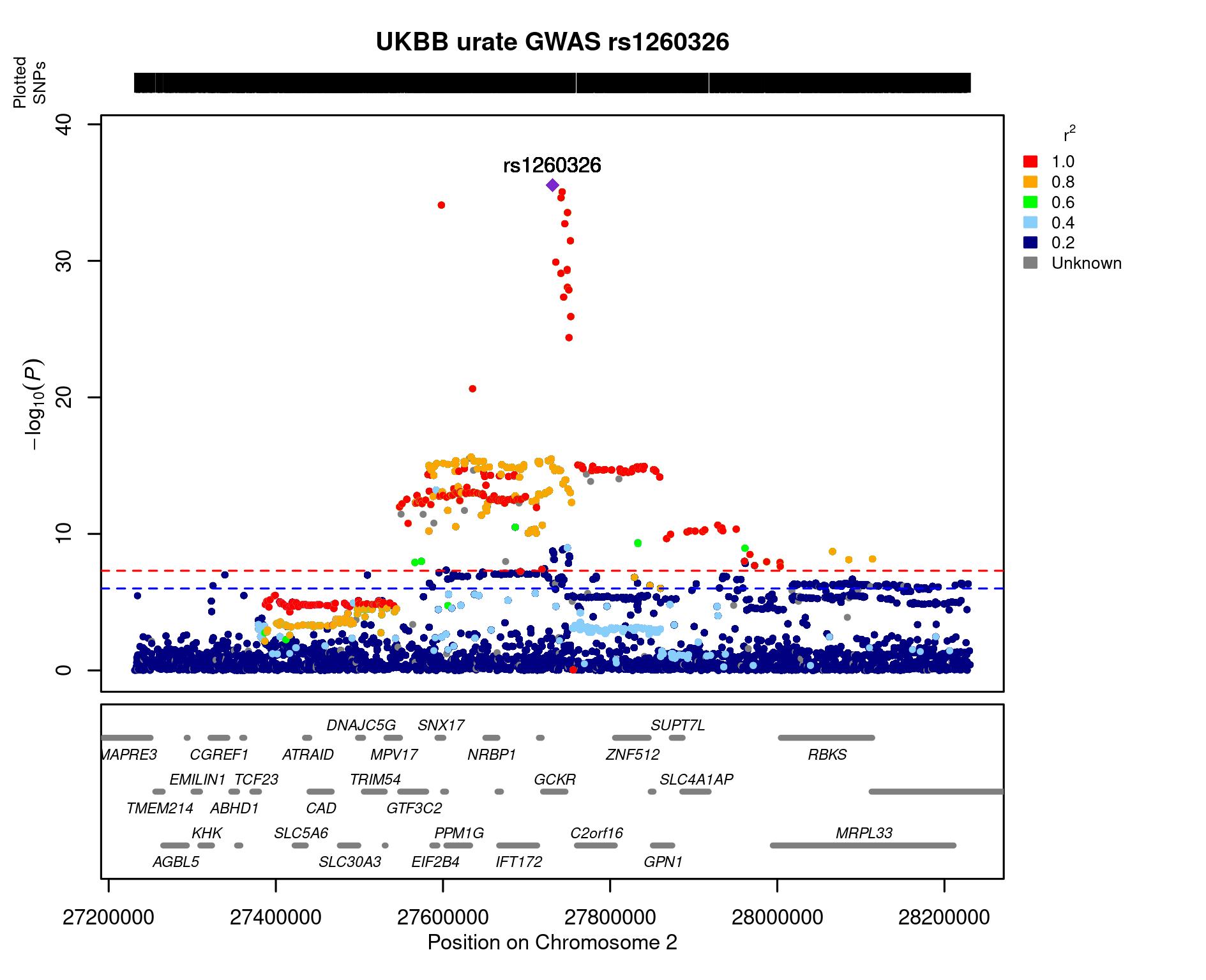
*GCKR* locus: lead shared SNP *GCKR* rs1260326.
2. *
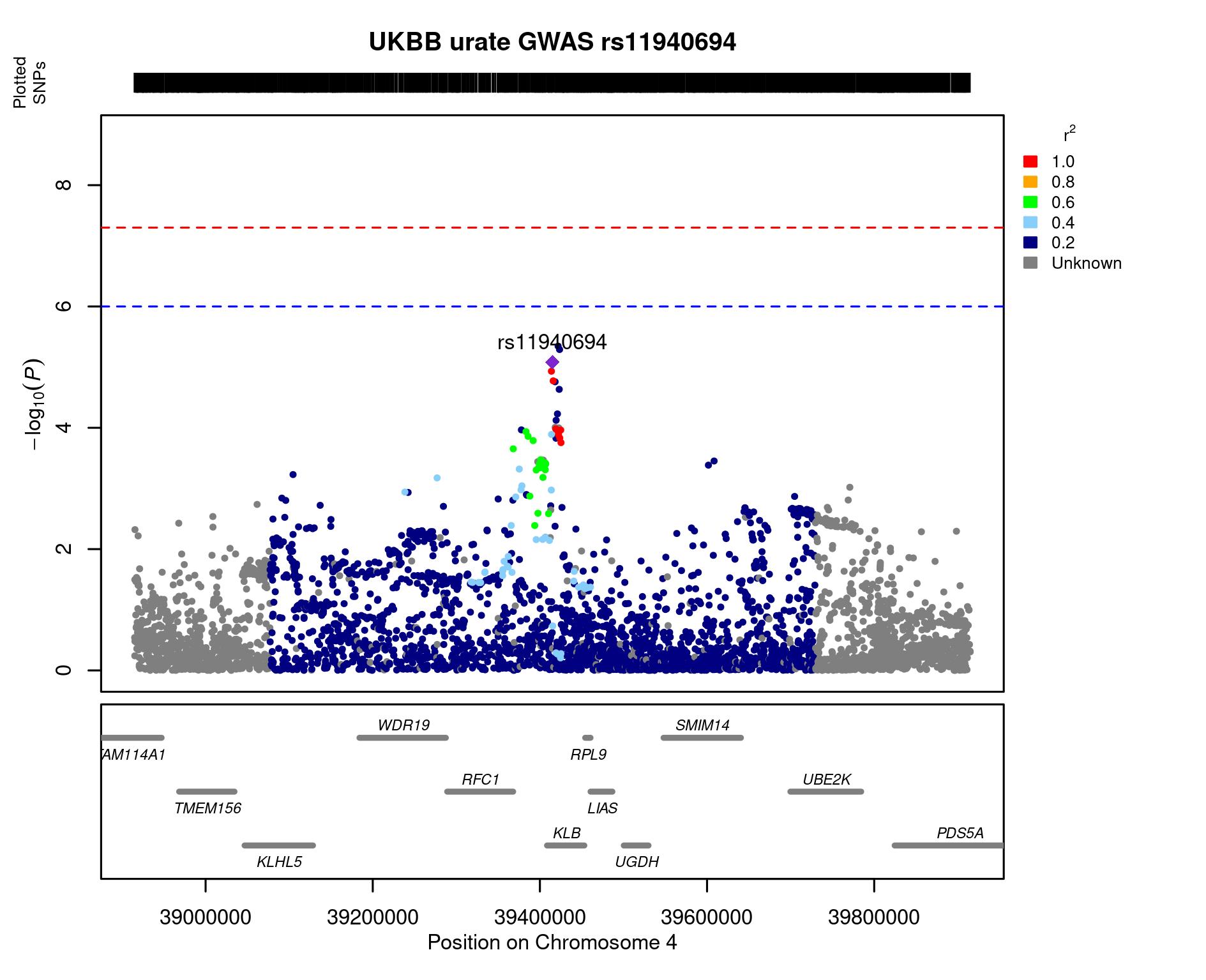
KLB* locus: lead shared SNP *KLB* rs11940694.
3.
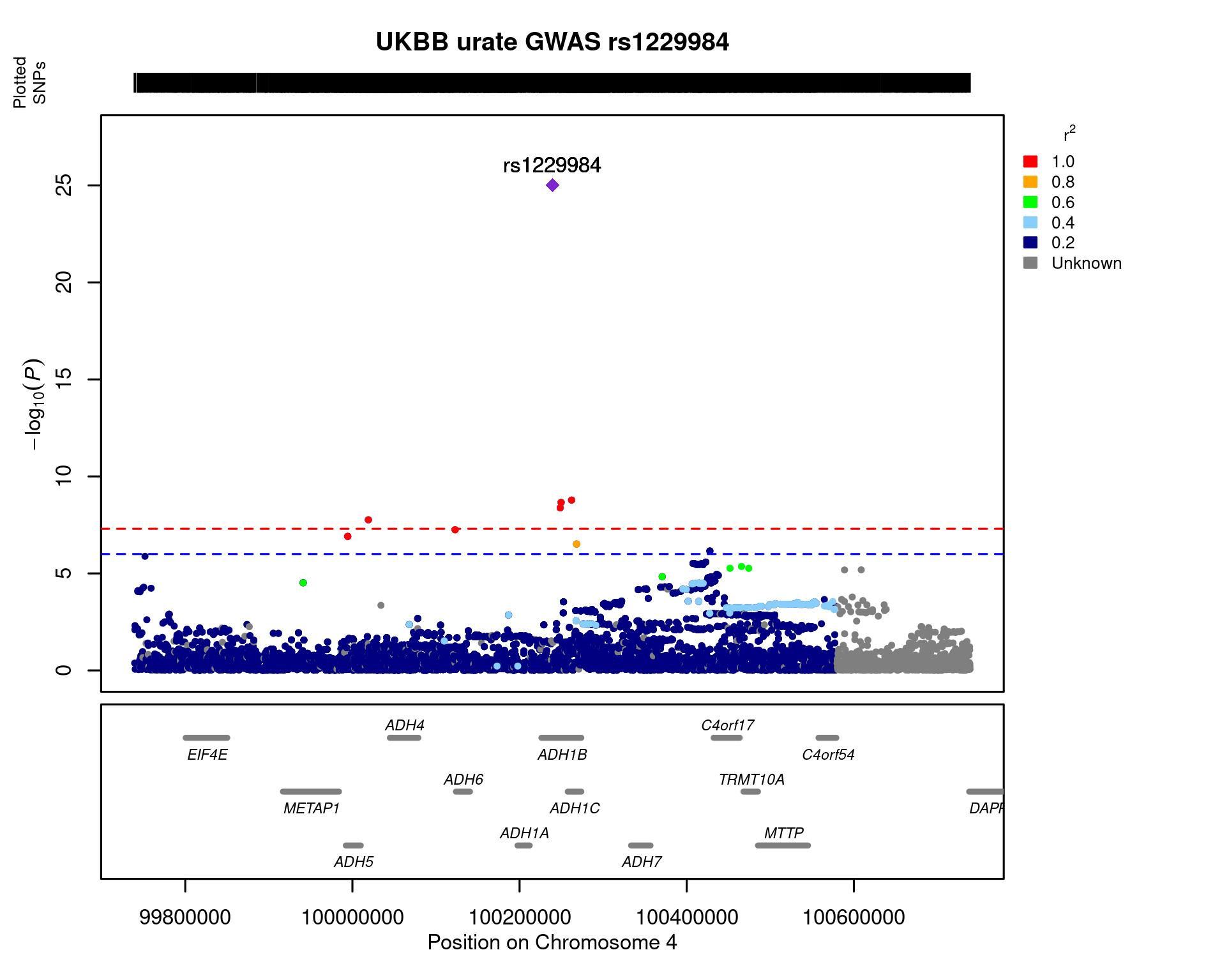
*ADH1B* locus: lead shared SNP *ADH1B* rs1229984.
4.
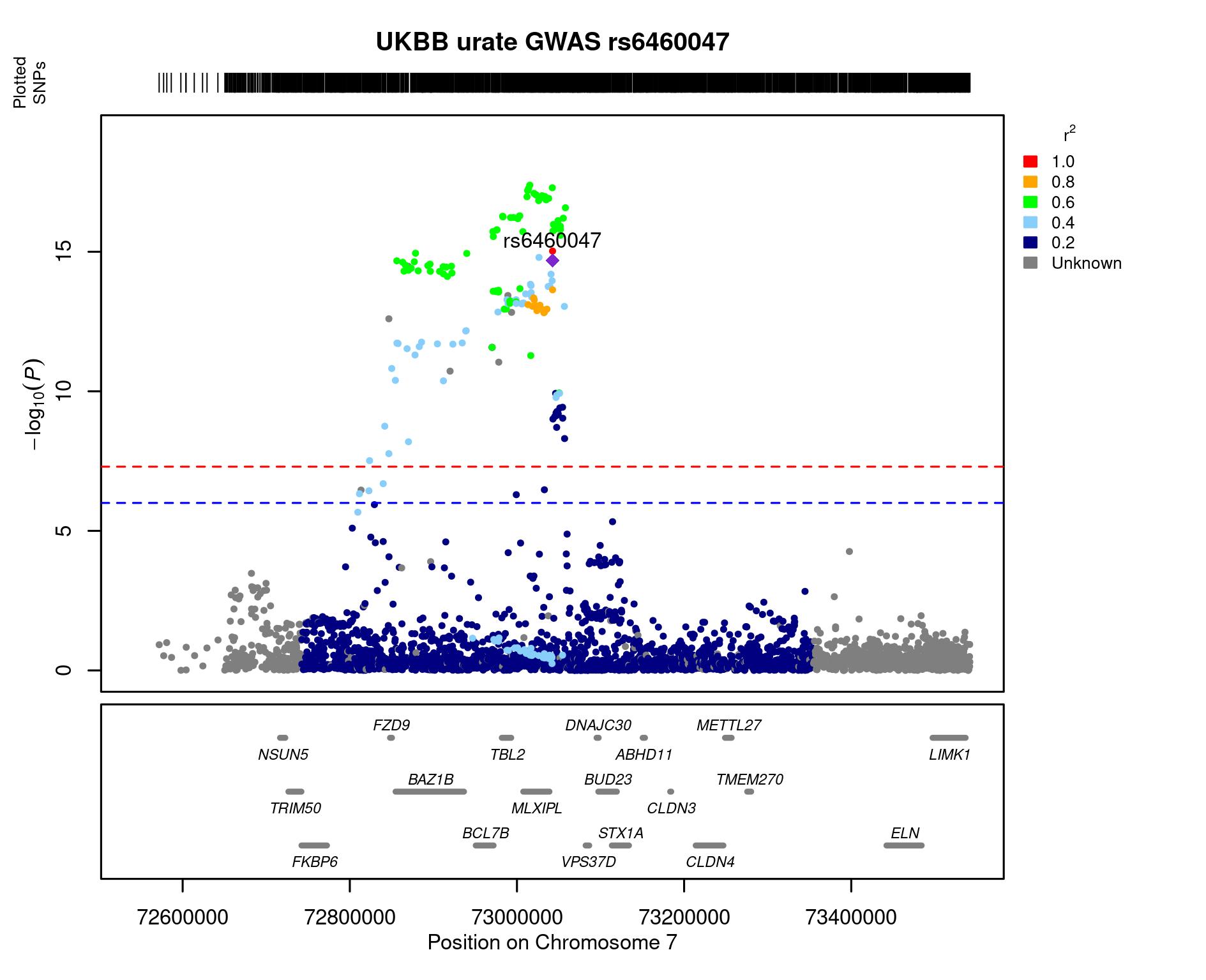
*MLXIPL* locus: lead shared SNP intergenic rs6460047.
5.
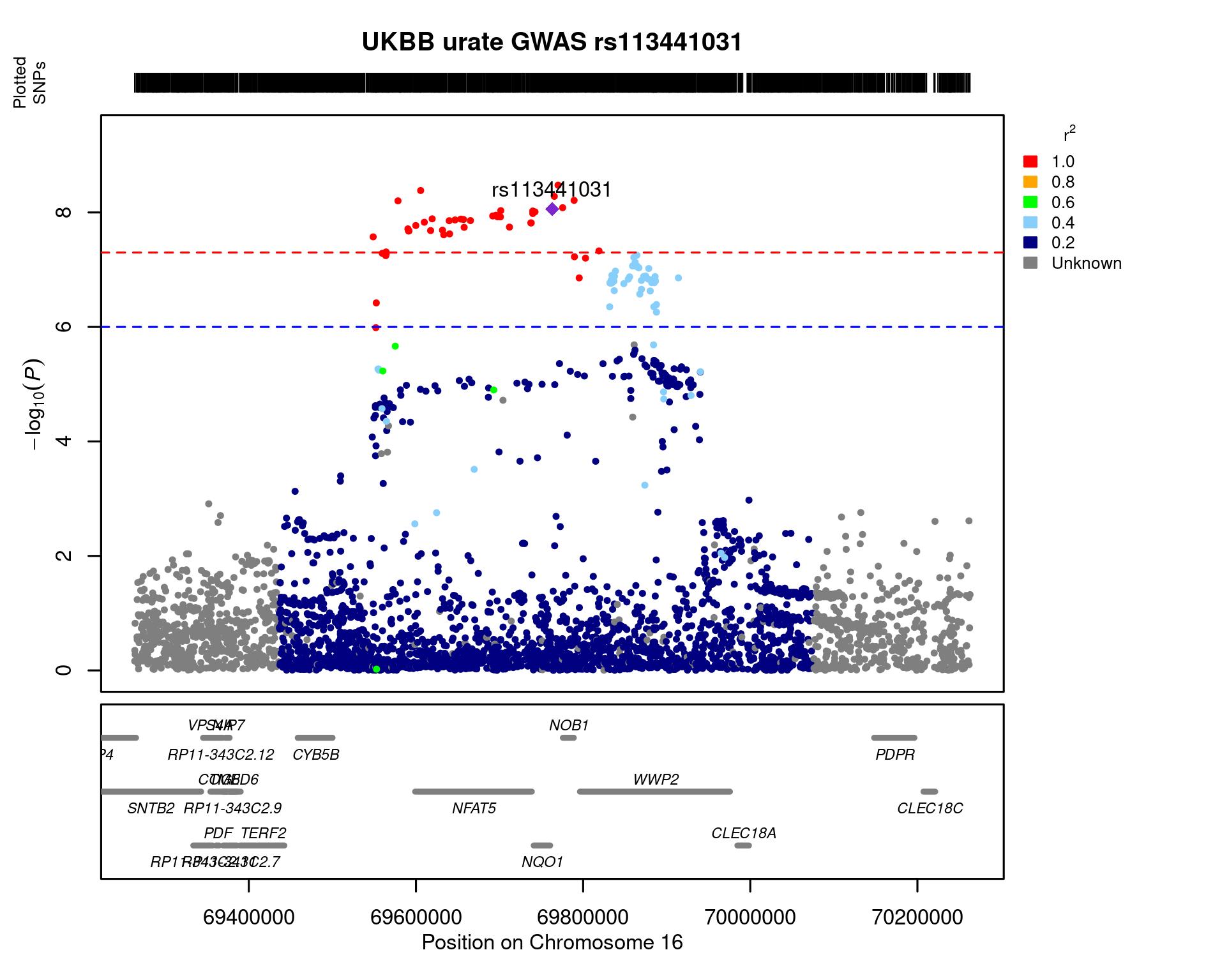
*NFAT5* locus: lead shared SNP *NQO1-DT* rs13441031.

**Figure S3. LocusZoom plots within UK Biobank for four gout loci included in interaction analysis.**

1.
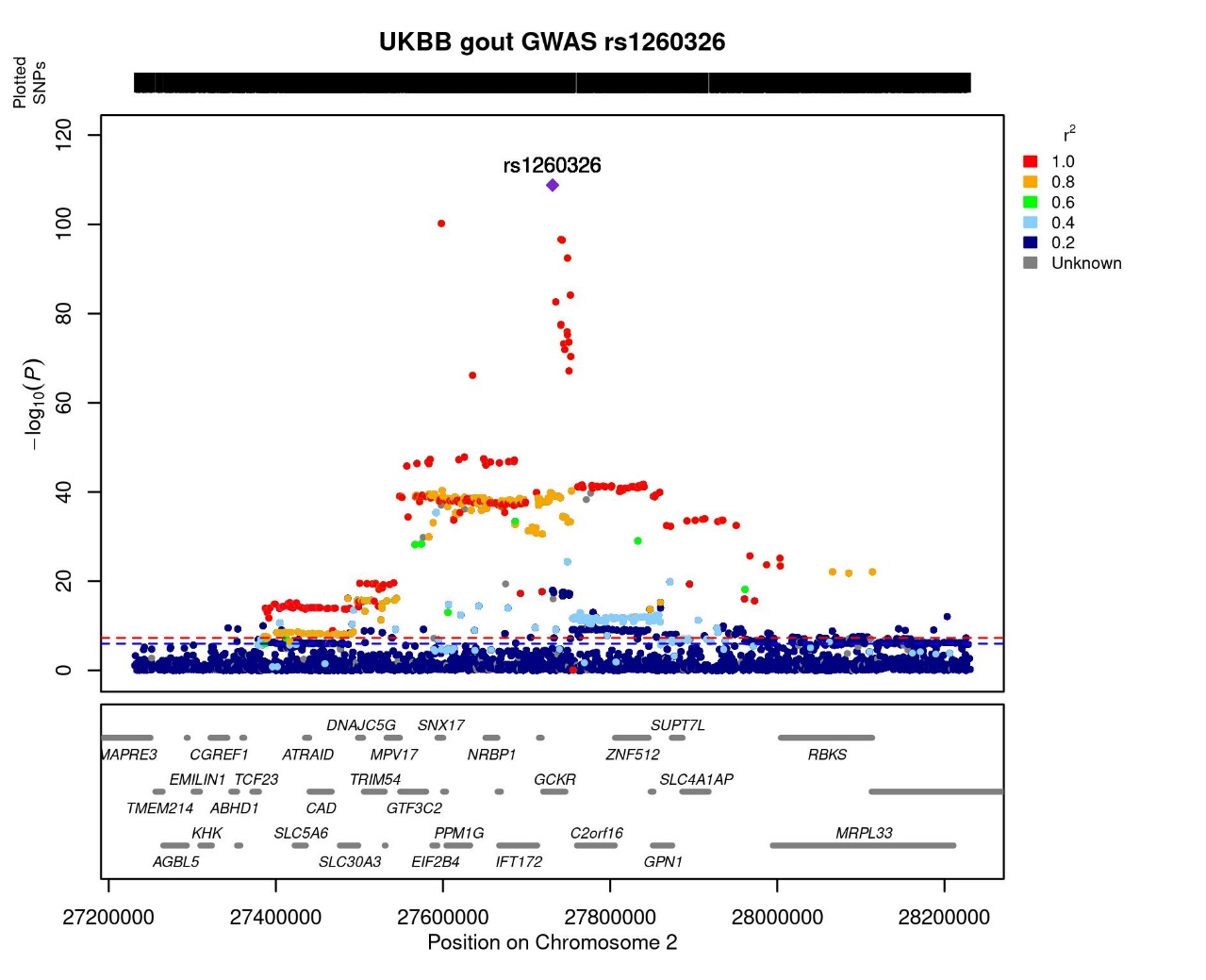
*GCKR* locus: lead shared SNP *GCKR* rs1260326.
2.
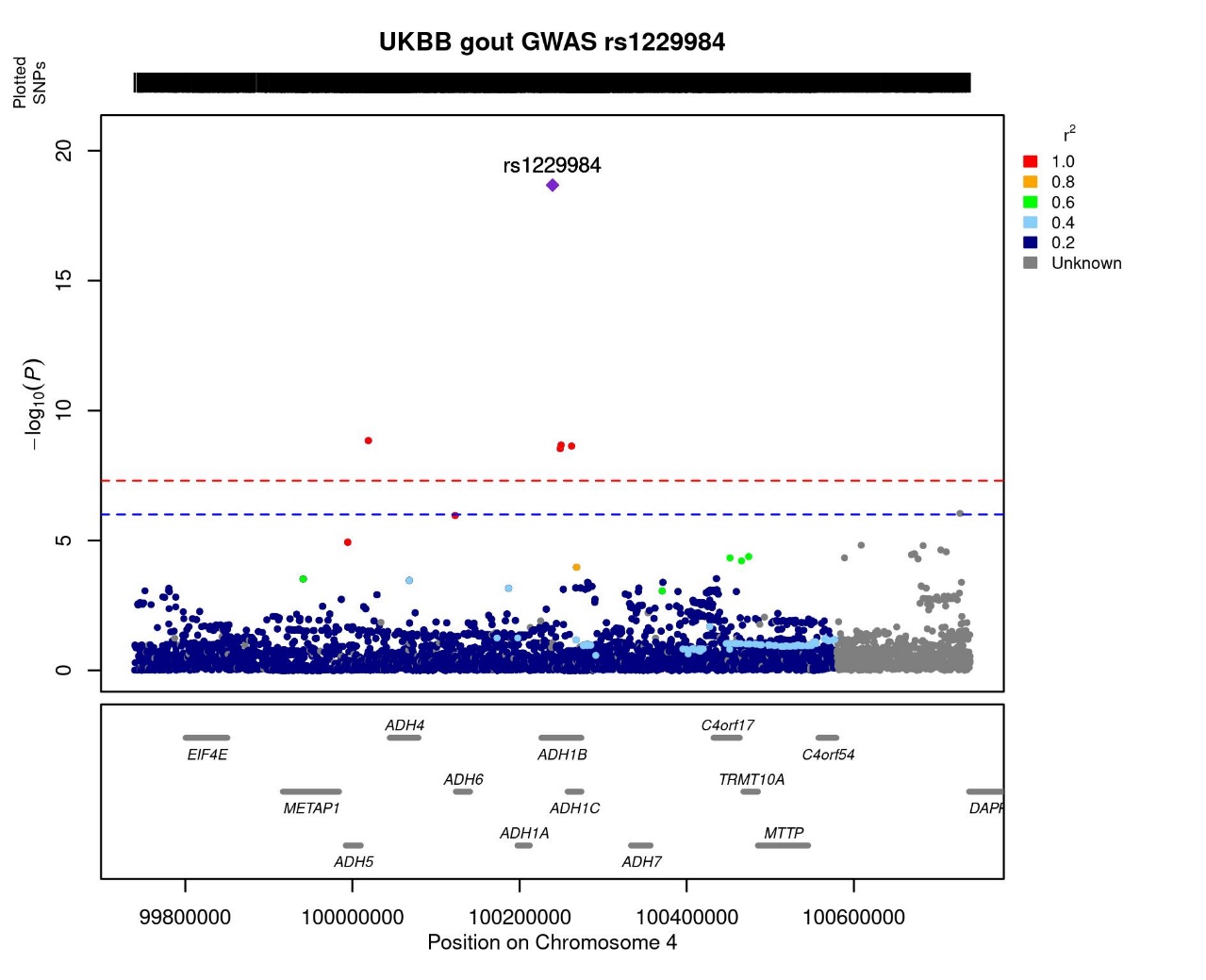
*ADH1B* locus: lead shared SNP *ADH1B* rs1229984.
3.
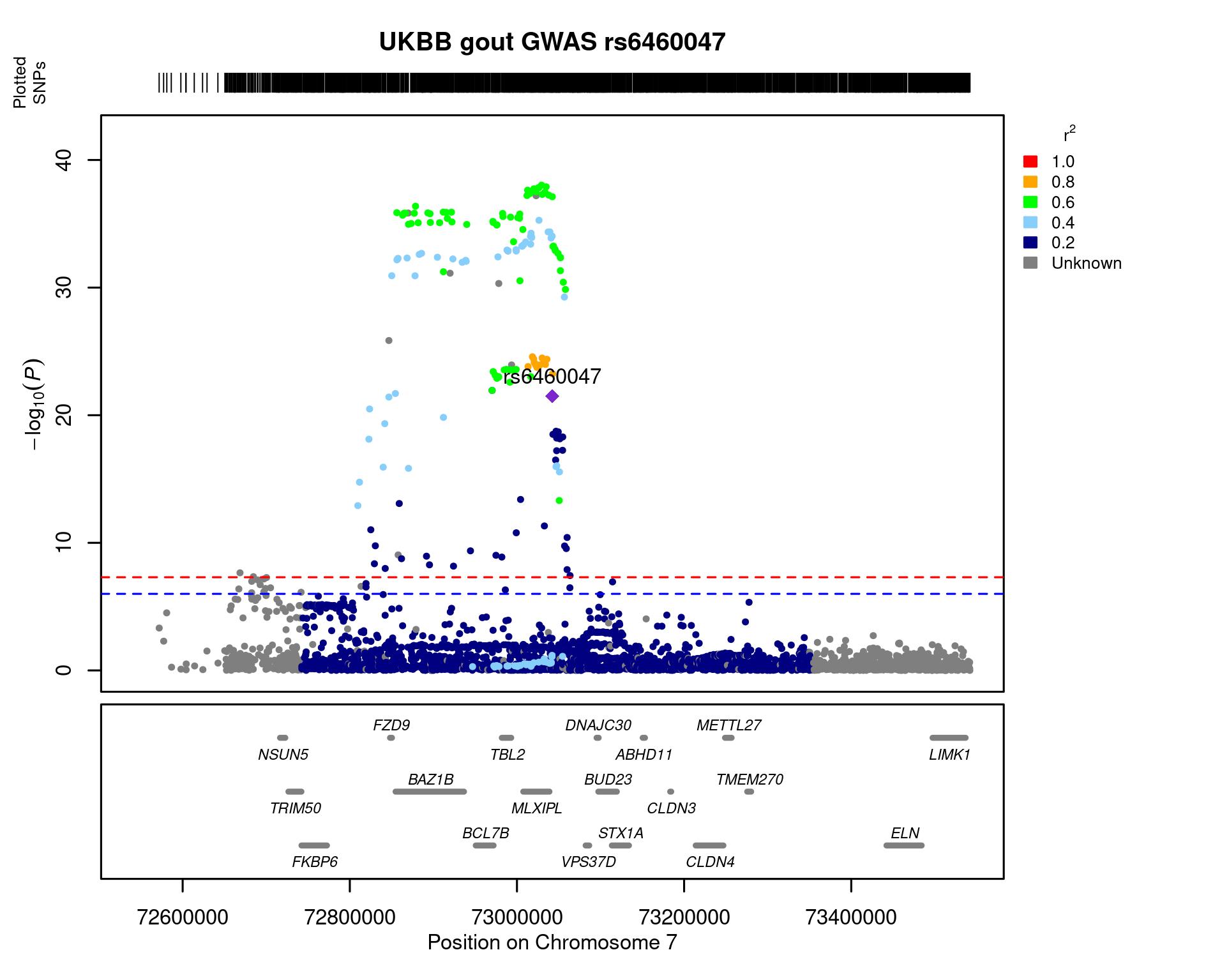
*MLXIPL* locus: lead shared SNP intergenic 6460047.
4.
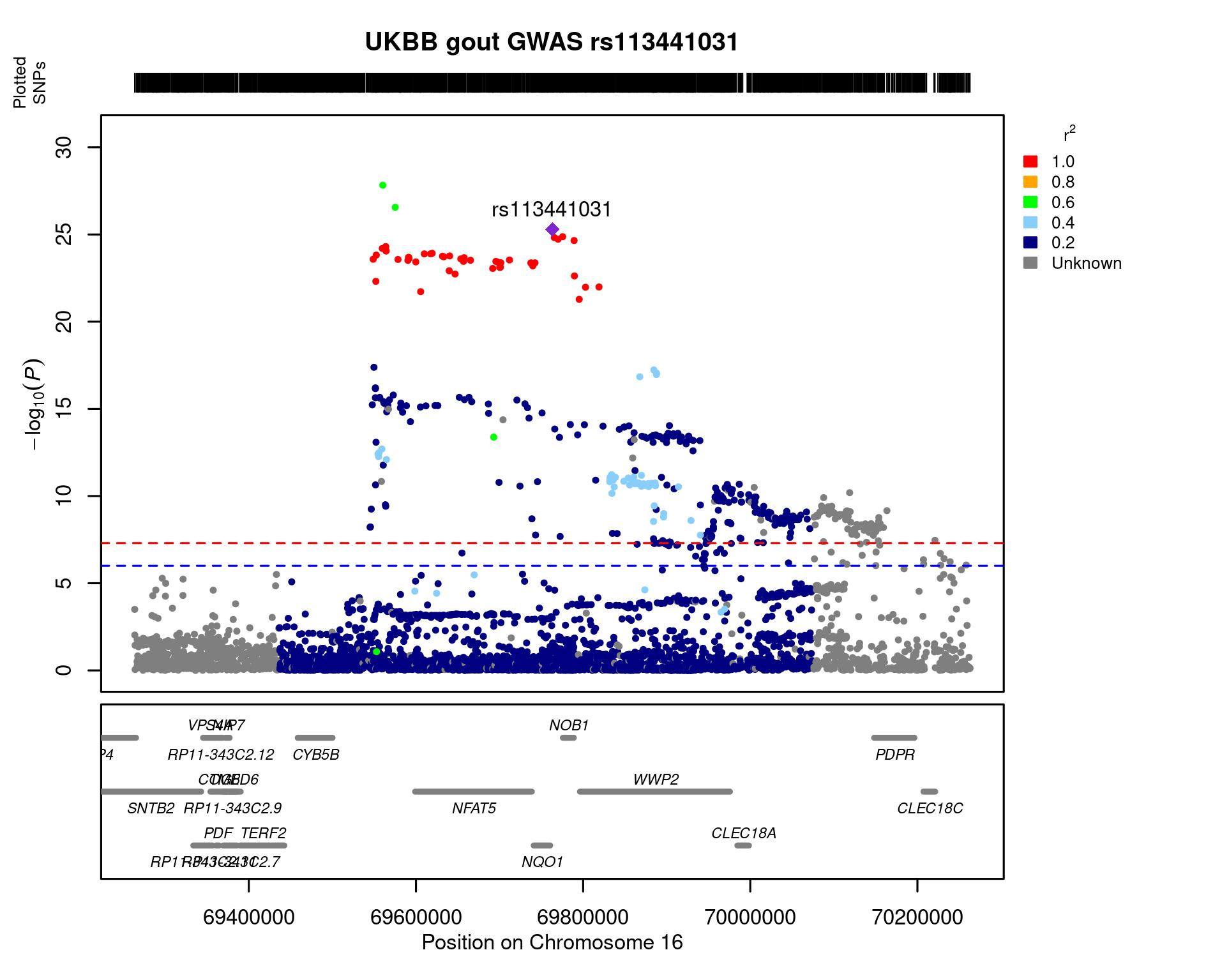
*NFAT5* locus: lead shared SNP *NQO1-DT* rs113441031.
